# Effectiveness of a Psychosocial Care Quality Improvement Strategy to Address Quality of Life in Patients With Cancer: The HuCare2 Stepped-Wedge Cluster Randomized Trial

**DOI:** 10.1001/jamanetworkopen.2021.28667

**Published:** 2021-10-14

**Authors:** Caterina Caminiti, Maria Antonietta Annunziata, Claudio Verusio, Carmine Pinto, Mario Airoldi, Marcello Aragona, Francesca Caputo, Saverio Cinieri, Paolo Giordani, Stefania Gori, Rodolfo Mattioli, Silvia Novello, Antonio Pazzola, Giuseppe Procopio, Antonio Russo, Giuseppina Sarobba, Filippo Zerilli, Francesca Diodati, Elisa Iezzi, Giuseppe Maglietta, Rodolfo Passalacqua

**Affiliations:** 1 Research and Innovation Unit, University Hospital of Parma, Parma, Italy; 2 Oncological Psychology Unit, National Cancer Institute, Aviano, Pordenone, Italy; 3 Department of Oncology, ASST Valle Olona, Busto Arsizio, Varese, Italy; 4 Medical Oncology Unit, Clinical Cancer Center, AUSL-IRCCS of Reggio Emilia, Reggio Emilia, Italy; 5 Second Medical Oncology Division, AOU Città della Salute e della Scienza of Turin, Turin, Italy; 6 Medical Oncology Unit, Department of Human Pathology of Adult and Evolutive Age, University of Messina, Messina, Italy; 7 Oncology Unit, Dei Colli Hospital, Naples, Italy; 8 Oncology Unit, San Antonio Perrino Hospital, Brindisi, Italy; 9 Medical Oncology Unit, Ospedali Riuniti Marche Nord, Pesaro and Fano, Italy; 10 Medical Oncology Division, IRCCS Sacro Cuore Don Calabria Hospital, Negrar di Valpolicella, Verona, Italy; 11 Department of Oncology, University of Turin, AOU San Luigi Orbassano, Italy; 12 Medical Oncology Unit, University-Hospital of Sassari, Sassari, Italy; 13 Department of Medical Oncology, National Cancer Institute of Milan, Milan, Italy; 14 Medical Oncology Unit, Department of Surgical, Oncological and Oral Sciences, University of Palermo, Palermo, Italy; 15 Oncology Unit, San Francesco Hospital, ATS Sardegna ASSL Nuoro, Nuoro, Italy; 16 Department of Oncology, ASP of Trapani, Trapani, Italy; 17 Medical Oncology Division, Department of Oncology, ASST of Cremona, Cremona, Italy

## Abstract

**Importance:**

Many patients with cancer who would benefit from psychosocial care do not receive it. Implementation strategies may favor the integration of psychosocial care into practice and improve patient outcomes.

**Objective:**

To evaluate the effectiveness of the Humanization in Cancer Care (HuCare) Quality Improvement Strategy vs standard care as improvement of at least 1 of 2 domains (emotional or social function) of patient health-related quality of life at baseline and 3 months. A key secondary aim included investigation of the long-term effect.

**Design, Setting, and Participants:**

HuCare2 was a multicenter, incomplete, stepped-wedge cluster randomized clinical trial, conducted from May 30, 2016, to August 28, 2019, in three 5-center clusters of cancer centers representative of hospital size and geographic location in Italy. The study was divided into 5 equally spaced epochs. Implementation sequence was defined by a blinded statistician; the nature of the intervention precluded blinding for clinical staff. Participants included consecutive adult outpatients with newly diagnosed cancer of any type and stage starting medical cancer treatment.

**Interventions:**

The HuCare Quality Improvement Strategy comprised (1) clinician communication training, (2) on-site visits for context analysis and problem-solving, and (3) implementation of 6 evidence-based recommendations.

**Main Outcomes and Measures:**

The primary outcome was the difference between the means of changes of individual scores in emotional or social functions of health-related quality of life detected at baseline and 3-month follow-up (within each group) and during the postintervention epoch compared with control periods (between groups). Long-term effect of the intervention (at 12 months) was assessed as a secondary outcome. Intention-to-treat analysis was used.

**Results:**

A total of 762 patients (475 [62.3%] women) were enrolled (400 HuCare Quality Improvement Strategy and 362 usual care); mean (SD) age was 61.4 (13.1) years. The HuCare Quality Improvement Strategy significantly improved emotional function during treatment (odds ratio [OR], 1.13; 95% CI, 1.04-1.22; *P* = .008) but not social function (OR, 0.99; 95% CI, 0.89-1.09; *P* = .80). Effect on emotional function persisted at 12 months (OR, 1.05; 95% CI, 1.00-1.10; *P* = .04).

**Conclusions and Relevance:**

In this trial, the HuCare Quality Improvement Strategy significantly improved the emotional function aspect of health-related quality of life during cancer treatment and at 12 months, indicating a change in clinician behavior and in ward organization. These findings support the need for strategies to introduce psychosocial care; however, more research is needed on factors that may maximize the effects.

**Trial Registration:**

ClinicalTrials.gov Identifier: NCT03008993

## Key Points

**Question:**

Does an implementation strategy integrating psychosocial care into cancer centers improve at least 1 of 2 domains (emotional or social function) of health-related quality of life?

**Findings:**

This stepped-wedge cluster randomized clinical trial included 762 patients with cancer at high risk for reduced quality of life. Health-related quality of life score improved from baseline to 3 months among participants in the Humanization in Cancer Care Quality Improvement Strategy arm vs the usual care arm, showing a statistically significant difference for emotional function, but not for social function.

**Meaning:**

This study suggests the use of an implementation strategy aiming to provide routine psychosocial care in cancer centers may be beneficial to patients; further investigation is required on factors that can maximize its effects.

## Teaser

This randomized clinical trial examines the use of psychosocial interventions in cancer treatment centers to improve patients’ health-related quality of life.

## Introduction

Individuals with cancer experience a wide range of psychosocial health needs, encompassing mental, emotional, social, and spiritual aspects of health.^1^ Frequent practical difficulties and information needs add to the cancer burden.^1,2^ These problems are associated with a decline in quality of life over time and thus should be the target of comprehensive care.^3,4^ To address these needs, a wide range of psychosocial interventions are available, including any activity aimed at ameliorating or reducing the influence of cancer on mental health and at improving patients' skills to cope with the demands of treatment and uncertainty of the disease outcome across the whole spectrum, from prediagnosis to palliative care and survivorship.^5^

Despite numerous clinical practice guidelines, many patients who might benefit from psychosocial interventions do not receive them.^3,5,6^ A survey of the International Federation of Psycho-oncology Societies^7^ including 25 countries concluded that the development and implementation of psycho-oncology was fragmented and undeveloped. In addition, in Europe, Italy in particular, these services were often regarded as not essential. Barriers to implementation may be related to personal characteristics of health care professionals, as well as to environmental and organizational factors.^8,9^Several of these barriers are modifiable,^8^ particularly using implementation strategies.^10,11,12^ These strategies should be tailored to potential obstacles,^13^ should be feasible, and should be effective.^14,15^

These considerations formed the basis for the Humanization in Cancer Care (HuCare) Quality Improvement Strategy (HQIS) aiming to integrate into practice 6 psychosocial interventions recommended by national and international guidelines^1,16,17,18^ selected by a multidisciplinary task force.^19^ Following the process for the development and evaluation of complex interventions of the Medical Research Council,^20^ the HuCare Study,^19^ conducted from 2008 to 2014, described the feasibility of the HQIS in 28 Italian cancer centers, obtaining a high level of adherence to all interventions (>85%). We then conducted this randomized clinical trial (HuCare2) to evaluate the effectiveness of the HQIS vs standard care in terms of improvement of health-related quality of life (HRQOL).

## Methods

### Study Setting and Design

This was a multicenter, incomplete (because data were not collected during implementation), stepped-wedge cluster randomized clinical trial in which the intervention strategy was sequentially carried out in 3 clusters of 5 centers each and in 3 equally spaced 4-month epochs.^21^ The study took place from May 30, 2016, to August 28, 2019. The study included an initial epoch when none of the centers used the intervention and a final epoch when all centers had implemented the strategy.^22,23^ Implementation epochs were randomly assigned. The intervention was applied at a cluster level—the unit of randomization—and assessed at an individual level (in the patients of each cluster) with a cross-sectional model (ie, patients were different in each epoch). Justifications for the design are provided in the protocol (Supplement 1).^21^ Approval for the study was obtained from the ethics committee of Cremona, the coordinating center, and from ethics committees of all participating sites, and written informed consent was provided by all patients. This study followed the Consolidated Standards of Reporting Trials (CONSORT) reporting guideline and its extensions (Patient Reported Outcomes Extension and Extension to Cluster Randomized Trials).^24^

Center recruitment was performed using a brief survey to ascertain motivation and the presence of prerequisites (eg, staff willingness to attend residential training, availability of a room dedicated to encounters with patients to provide information, and availability of a psychologist in the ward). The number of centers was determined according to feasibility, ensuring representativeness of size (number of beds) and geographic location (north, center-south, islands). Centers that had participated in the HuCare feasibility study were not eligible for this trial.

Before trial initiation, a pilot study was performed at the Cremona cancer center on a consecutive sample of 13 patients who accessed the facility over 2 weeks to measure the feasibility and acceptability of questionnaire administration using a computer tablet. The findings of this pilot study led to changes in the protocol, including removal of the upper age limit, extension to hormonal therapy and immunotherapy, and exclusion of patients enrolled in other trials, implying the use of patient-reported outcomes.

Eligible individuals were outpatients older than 18 years with cancer of any type and stage who were consecutively accessed in the center during a 2-week index period, had received the diagnosis within the previous 2 months, were about to start a new medical cancer treatment, had expected survival of more than 3 months, had good comprehension of the Italian language, and had signed the informed consent form. We excluded patients who had received previous chemotherapy or other medical cancer treatment; were recruited in a previous epoch of the study; were currently participating in other trials, implying the completion of patient-reported outcomes; were hospitalized; were receiving psychiatric treatment; were affected by mental or psychiatric disorders due to cancer or coexisting illness that were interfering with the state of consciousness or impeding judgment; or were unable to complete the questionnaire or ensure participation in the 3-month follow-up.

### Intervention and Outcomes

The HQIS has been described elsewhere.^19^ It consists of the provision of support activities to the centers aiming to introduce 6 evidence-based psychosocial recommendations—1 targeting clinicians and 5 targeting patients.^21^ The strategy lasts 16 weeks and comprises 3 phases (Supplement 1). In phase 1, medical and nursing staff attend communication skills training designed according to literature-reported indications (recommendation 1). In phase 2, center support is provided by the improvement team, composed of external personnel (sociologist, psychologist, and research nurse), during on-site visits. According to the plan-do-study-act cycle,^25^ problems and solutions discussed during one site visit are used to introduce change and inform the following visit. A description of the instruments used can be found in the eMethods in Supplement 2. In phase 3, centers independently implement the 5 psychosocial interventions targeting patients (recommendations 2-6): provision of a question prompt list (validated set of possible questions that patients may ask the oncologist); assignment of a specialist nurse; screening for psychological distress and social needs, with activation of appropriate services; and access to the point of information and support, which is a library for patients and their families managed by trained nurses. When centers were in the control epoch, patients received usual care (ie, what was done in routine practice).

The primary outcome was the difference between the means of changes of individual scores in emotional function (EF) or social function (SF) of HRQOL detected at baseline and 3-month follow-up (within each group) and during the postintervention epoch compared with control periods (between groups). These 2 functions were chosen because they are mostly affected by psychosocial interventions, following evidence-based guidelines.^26^ The corresponding timing (ie, time of effect measurement) was selected because, during periods of active treatment, improvement from baseline is more likely to be observed.^26^ Health-related quality of life was measured with the validated Italian version of the European Organisation for Research and Treatment of Cancer Quality of Life Questionnaire, version 3 (EORTC QLQ-C30).^27^ The questionnaire was self-administered using a touch-screen tablet at baseline and 3 months after enrollment.

The secondary aim was to investigate whether the strategy had an effect on mood disorders, defined as a cutoff score greater than 7 on the Hospital Anxiety and Depression Scale–Depression (HADS-D) scale^28,29^ over the long-term (up to 1 year), overall HRQOL, and specific domains. Subgroup analyses were performed to obtain information about treatment effect modifiers and subsequently define how to implement more targeted interventions. Process indicators were also measured: frequency of clinical staff (oncologists and nurses) who completed training and proportion of patients who, at 3-month follow-up, experienced a reduction in their baseline unmet needs detected with the Needs Evaluation Questionnaire, a self-administered instrument with 23 dichotomous items divided into 5 areas.^30,31,32,33^

### Statistical Analysis

The number of participants was defined following the methods for incomplete, cross-sectional, stepped-wedge cluster randomized trials,^34,35,36,37^ considering (1) 3 clusters, each comprising 5 centers of equal size (capacity of enrollment per week); (2) a mean expected difference lying between 3 and 8 points of at least 1 function (social or emotional), with values indicated by Cocks et al^38^; (3) an intraclass correlation coefficient equal to 0.80, as reported in 2 articles^39,40^; (4) the Wald test, with time as the fixed effect and the cluster as the random effect^34^; (5) a power of 80% and 2-tailed α threshold of 5%; and (6) a dropout rate of 20% at follow-up.^41^ Applying the Stata/MP 11.2 *steppedwedge* procedure,^36^ we calculated an overall sample size of 720 patients, meaning 60 patients in each cluster for every detection epoch.

Each cluster was defined including 5 centers located in the same geographic area (north, center-south, islands) to facilitate the work of the improvement team. Randomization to define the intervention’s implementation sequence was performed through SAS software by the trial statistician (E.I.), who informed centers of their assigned implementation period with a 4-week notice. Blinding was ensured both for patients, who were not informed of the study epoch (control or postintervention), and for the statistician, who used anonymized data and encrypted identification codes for the study epochs. The nature of the intervention precluded blinding for clinical staff.

Patients entered data anonymously using tablet computers that included control checks. For the principal analysis of effectiveness, we considered an intention-to-treat population, composed of all clusters according to randomization and all eligible patients with HRQOL assessments at baseline and 3-month follow-up. Differences of HRQOL values between the 2 groups were analyzed using a binomial β regression model, as suggested by different authors,^42,43^ owing to the asymmetric value distribution. This model also enabled an estimate of the strategy’s effect in terms of odds ratio (OR), the preferred measure by oncologists for its more immediate interpretation and greater usefulness in clinical practice compared with absolute values.^42^ For the binomial β model analysis, responses were transformed into a scale (0, 1) by using the formula Y-a / b-a, where a is the lowest and b is the highest possible score and Y is the observed response. Precision coefficient was measured using the Log-phi parameter. The demographic and clinical variables that correlated with the outcome at *P* < .20 in the univariate analysis were included in the regression model.

Because the study was a cross-sectional, stepped-wedge cluster randomized clinical trial (with an implementation period), the final model was adjusted extending the basic Hussey and Hughes model^34,44^ to include a fixed interaction between intervention status and implementation period (first, second, or third).

Concerning the secondary outcomes, intention-to-treat analyses were performed. First, long-term variables were entered into the binomial β model as covariates in the model for the principal analysis. Second, the EORTC QLQ-C30 global scales and the other domain scales were represented as observed response Y in the binomial β model, and the covariates were those used in the primary analysis. Third, to examine whether the HQIS had any effect on mood disorders, general linear modeling with findings significant at *P* < .05 was used.

To assess whether the intervention modified associations of the primary outcome measure with candidate variables (subgroup analysis), the binomial β model was performed separately for each predictor. In these analyses, the difference in HRQOL values between preintervention and postintervention scores served as the dependent or outcome variables. The interaction terms of each candidate predictor with the randomized treatment groups dummy (HQIS intervention vs control) served as the independent predictor, along with constituent main effects.

Concerning reduction of unmet needs (process indicator), the χ2 test was used. Data were processed with SAS, software STATA/SE version 11.0 (StataCorp LLC) and R-cran, version 3.5.3 (R Foundation for Statistical Computing).

## Results

### Participants and Centers

**Figure 1.**
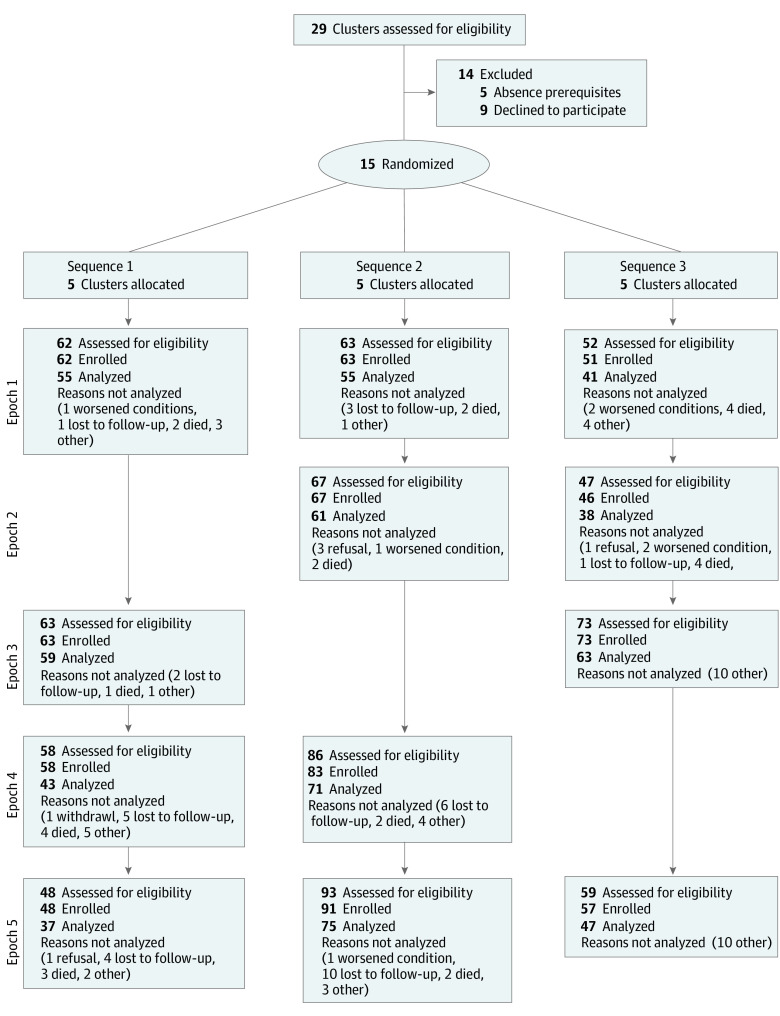
Stepped-Wedge Cluster Randomized Clinical Trial by Allocated Sequence and Epochs

Cluster, center, and participant characteristics are provided in Figure 1. A total of 762 patients were enrolled: 400 in the HQIS arm and 362 in the usual care arm. At baseline, the mean (SD) age was 61.4 (13.1) years, 475 (62.3%) were women, 287 (37.3%) were men, Eastern Cooperative Oncology Group Performance Status was 0 in 577 patients (75.7%), indicating the ability to carry on all predisease performance without restriction The most prevalent cancer type was genitourinary cancer (272 [35.7%]); 194 patients (25.5%) had metastases and 660 patients (86.6%) were receiving chemotherapy. A total of 230 of 756 patients (30.4%) had a HADS-D score less than 8 (no disorder) and 123 of 756 patients (16.3%) a score greater than 22 (severe disorder). A total of 669 of 761 patients with data available (87.9%) had at least 1 psychosocial need. Other baseline characteristics were similar in the 2 groups (Table 1), except for differences in type of cancer, which are due to chance.

**Table 1.** Summary of Baseline Characteristics by Groups

Characteristic	No. (%)		
	HQIS (n = 400)	Usual care (n = 362)	Total (n = 762)
Sex			
Female	227 (56.8)	248 (68.5)	475 (62.3)
Male	173 (43.3)	114 (31.5)	287 (37.7)
Age, mean (SD), y	62.5 (12.8)	60.1 (13.3)	61.4 (13.1)
Marital status			
Married/living with partner	312 (80.3)	288 (79.6)	609 (79.9)
Other	88 (22.0)	74 (20.4)	162 (21.3)
Educational level			
Primary education or less	175 (43.8)	185 (51.1)	360 (47.2)
High school	192 (48.0)	128 (35.4)	320 (42.0)
Graduate school	33 (8.3)	49 (13.5)	82 (10.8)
ECOG PS			
0	300 (75.0)	277 (76.5)	577 (75.7)
1	82 (20.5)	58 (16.0)	140 (18.4)
2	4 (1.0)	11 (3.0)	15 (2.0)
3	1 (0.3)	5 (1.4)	6 (0.8)
4	2 (0.5)	5 (1.4)	7 (0.9)
Missing	11 (2.8)	6 (1.7)	17 (2.2)
Treatment^a^			
Chemotherapy	362 (90.5)	298 (82.3)	660 (86.6)
Molecular target drugs	48 (12.0)	41 (11.3)	153 (20.1)
Hormone therapy	20 (5.0)	65 (18.0)	85 (11.2)
Immunotherapy	12 (3.0)	7 (1.9)	19 (2.5)
Presence of metastases	118 (29.5)	76 (21.0)	194 (25.5)
Type of cancer			
Breast	107 (26.8)	165 (45.6)	272 (35.7)
Colorectal colon	72 (18.0)	63 (17.4)	135 (17.7)
Lung	91 (22.8)	36 (9.9)	127 (16.7)
Head and neck	27 (6.8)	10 (2.8)	37 (4.9)
Other site	23 (5.8)	12 (3.3)	35 (4.6)
Stomach and esophagus	17 (4.3)	16 (4.4)	33 (4.3)
Pancreas	18 (4.5)	15 (4.1)	33 (4.3)
Gynecological	17 (4.3)	16 (4.4)	33 (4.3)
Urinary tract	9 (2.3)	19 (5.2)	28 (3.7)
Prostate	9 (2.3)	6 (1.7)	15 (2.0)
Liver	9 (2.3)	3 (0.8)	12 (1.6)
Blood	1 (0.3)	1 (0.3)	2 (0.3)
HADS-D^b^			
Total score, mean (SD)	12.1 (8.2)	14.2 (8.4)	13.1 (8.3)
<8 points	139 (35.1)	91 (25.3)	230 (30.4)
≥22 points	50 (12.6)	73 (20.3)	123 (16.3)
Missing	4 (1.0)	2 (0.6)	6 (0.8)
NEQ^c^			
≥1 Psychosocial need	337 (84.5)	332 (91.7)	669 (87.9)
Missing	1 (0.3)	0	1 (0.1)

Abbreviations: ECOG PS, Eastern Cooperative Oncology Group Performance Status; HADS-D, Hospital Anxiety and Depression Scale–Depression; HQIS, Humanization in Cancer Care Quality Improvement Strategy; NEQ, Needs Evaluation Questionnaire.

^a^ Some patients received more than 1 treatment.

^b^ HQIS, n = 396; usual care, n = 360.

^c^ HQIS, n = 399; usual care, n = 362.

Twenty-nine oncology wards showed interest; 5 were excluded because they lacked the necessary prerequisites, and 9 eventually declined participation. Reasons for the refusal included a psychosocial program already in place, difficulties in ensuring staff participation in residential training, or lack of support from hospital management. In epoch 1 (September 2016 to January 2017), no center received the intervention, in epoch 2 (February to May 2017), the intervention was provided to cluster 1, in epoch 3 (June to November 2017) to cluster 2, in epoch 4 (December 2017-April 2018) to cluster 3. In epoch 5 (May 2018-August 2018), all centers were implementing the psychosocial recommendations. Epochs 3 and 4 were extended because they included a holiday season.

### Outcomes

**Figure 2.**
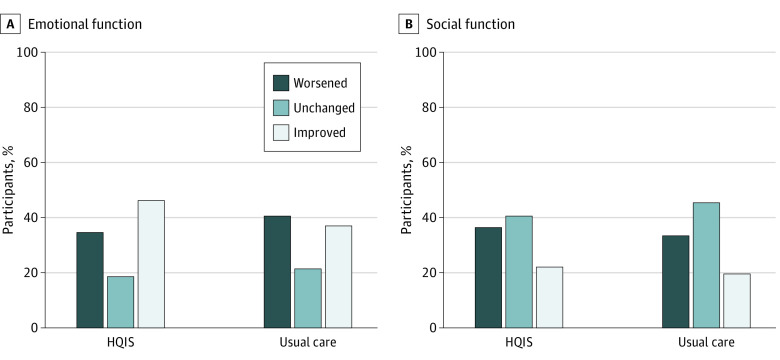
Percentage of Patients Who Exhibited Change in the Emotional or Social Functions of Health-Related Quality of Life at 3-Month Follow-up vs Baseline

Data analysis was conducted on 647 patients (332 HQIS and 315 usual care) without missing data. The HRQOL score improved by any amount from baseline to 3 months among more participants in the HQIS arm than in the usual care arm both for EF (45% vs 37%), and for SF (22% vs 20%), but the score worsened among fewer patients for EF (HQIS, 35% vs usual care, 41%) (Figure 2, eTable 1 in Supplement 2).

Results for the primary outcome are reported in Table 2. The HQIS intervention significantly improved EF (mean difference, 1.56; OR, 1.13; 95% CI, 1.04-1.22; *P* = .008), but not SF (mean difference, 2.21; OR, 0.99; 95% CI, 0.89-1.09; *P* = .80). The effect on EF was significantly larger for younger patients and for those with fewer needs at 3 months compared with baseline. Furthermore, we observed that the implementation period was statistically significant, indicating different effects linked to the cluster, with the smallest benefits for cluster 3.

**Table 2.** Primary Outcome: Effects of HQIS on the Emotional and Social Functions of HRQOL

Variable	Emotional function^a^				Social function^b^			
	Univariate analysis	Multivariate analysis			Univariate analysis	Multivariate analysis		
	*P* value^c^	β	OR (95% CI)	*P* value^c^	*P* value^c^	β	OR (95% CI)	*P* value^c^
Intercept	NA	−0.392	0.68 (0.43 to 0.93)	.002	NA	−0.140	0.87 (0.70 to 1.03)	.10
HQIS vs Usual care	NA	0.121	1.13 (1.04 to 1.22)	.008	NA	−0.013	0.99 (0.89 to 1.09)	.80
HQIS implementation, epoch 1 vs 3 (cluster 1)	NA	0.133	1.14 (1.03 to 1.26)	.02	NA	−0.067	0.94 (0.81 to 1.06)	.30
HQIS implementation, epoch 2 vs 3 (cluster 2)	NA	0.158	1.17 (1.07 to 1.27)	.003	NA	0.056	1.06 (0.95 to 1.17)	.33
Age, y	.008	−0.004	1.00 (0.99 to 1.00)	.02	.39	NA	NA	NA
Male sex	.08	0.069	1.07 (0.98 to 1.16)	.13	.83	NA	NA	NA
Married	.62	NA	NA	NA	.19	0.092	1.10 (0.99 to 1.21)	.10
Educational level (more primary)	.62	NA	NA	NA	.22	NA	NA	NA
No metastases	.49	NA	NA	NA	.87	NA	NA	NA
Treatmentother than chemotherapy	.46	NA	NA	NA	.13	0.123	1.13 (1.00 to 1.27)	.07
ECOG PS	.98	NA	NA	NA	NA	NA	NA	NA
Met needs^d^	.001	0.175	1.19 (1.10 to 1.28)	<.001	.01	0.137	1.15 (1.05 to 1.25)	.009

Abbreviations: β, regression coefficient; ECOG PS, Eastern Cooperative Oncology Group Performance Status; HQIS, Humanization in Cancer Care Quality Improvement Strategy; HRQOL, health-related quality of life; NA, not applicable; OR, odds ratio.

^a^ Log-phi (SE), −2.778 (0.065).

^b^ Log-phi (SE), −2.511 (0.063).

^c^ *P* value of the binomial β regression model.

^d^ At least 1 met need.

Concerning secondary outcomes, the HQIS’s long-term effect was confirmed for EF at 12 months (OR, 1.05; 95% CI, 1.00-1.10; *P* = .04). No effect was detected for mood (HADS-D), the EORTC QLQ-30s global scale, and most remaining scales (eTables 2, 3, and 4 in Supplement 2). Subgroup analysis was performed considering candidate effect modifiers of EF on which the HQIS had a significant effect. None of the variables exhibited a statistically significant association (eTable 5 in Supplement 2).

Regarding the 2 process indicators, a high level of course attendance by clinical staff was observed (299 of 356 [84%]) and a greater reduction in the HQIS arm at 3-month follow-up was recorded in all 5 need areas (Table 3). Regarding the latter indicator, the difference was always statistically significant, with the OR at least 2 (ie, patients in the intervention group were twice as likely to have their needs met with compared with patients in the usual care group) (Table 3).

**Table 3.** Secondary Outcome: Percentage of Patients Who Exhibit Unmet Needs by Area of Needs Evaluation Questionnaire

Variable	No. (%)		OR (95% CI)
	HQIS	Usual care	
**Baseline**			
No.	399^a^	362	
Information needs	277 (69.4)	289 (79.8)	NA
Needs related to assistance/care	83 (20.8)	137 (37.8)	NA
Material needs	167 (41.9)	227 (62.7)	NA
Relational needs	229 (57.4)	252 (69.6)	NA
Needs for psychoemotional support	214 (53.6)	242 (66.9)	NA
**Follow-up**			
No.	332	315	
Information needs	173 (52.1)	234 (74.3)	2.65 (1.88-3.76)
Needs related to assistance/care	31 (9.3)	81 (25.7)	3.35 (2.11-5.44)
Material needs	108 (32.5)	182 (57.8)	2.83 (2.03-3.96)
Relational needs	81 (24.4)	185 (58.7)	4.40 (3.11-6.26)
Needs for psychoemotional support	146 (44.0)	207 (65.7)	2.43 (1.75-3.40)

Abbreviations: HQIS, Humanization in Cancer Care Quality Improvement Strategy; NA, not applicable; OR, odds ratio.

^a^ One missing response.

## Discussion

To our knowledge, this randomized clinical trial is the first report that a quality improvement strategy aimed at integrating evidence-based psychosocial care interventions into practice significantly improved EF of HRQOL in patients during cancer treatment. The effect persisted at 12 months, suggesting that once the HQIS strategy is learned and implemented, a lasting change occurs in clinical staff behavior and in ward organization.

These positive results support the need for strategies to introduce psychosocial care capable of addressing the multiple obstacles and barriers that may hinder implementation.^3,9^ In particular, our study demonstrates the effectiveness of a system-based approach, which implies organizational change requiring collaboration and commitment across hospital departments, disciplines, and individual clinicians.^45^ Despite the potential advantages of system-based interventions, the review by Sanson-Fisher et al^45^ of publications in 5 top-ranking journals in the field found that only the HuCare feasibility study^19^ met all 4 criteria for evaluating system-based change. Furthermore, we confirm the conclusions of a recent survey of 102 German oncologists^46^ in which physicians’ personal commitment to psycho-oncology was related to the integration of psycho-oncological aspects into patient treatment. The authors emphasize the importance of specific communication skills training, which was a central part of the HQIS. In the same survey, the perceived reluctance of patients to discuss their distress or patients’ assertion of not being distressed was seen by oncologists as a reason not to further pursue the topic. In this view, the HQIS comprised interventions aimed at encouraging patients to communicate openly with clinicians, build a trust relationship with them, and obtain needed information.

### Limitations

The trial has limitations. Although statistically significant, the difference in the mean change in HRQOL between the 2 groups was trivial—lower than the 3-point value that has been defined as the minimal important difference.^38^ There may be a number of reasons for this small difference, all requiring further investigation. First, randomized clinical trials may not be the best way to answer questions about the effects of interventions in complex systems, because the strict rules entailed by the design do not enable adjustment to the needs of different centers.^47^ Second, although a stepped-wedge cluster design provided greater trial efficiency, the 2-year period imposed by economic constraints did not allow us to observe all 3 clusters for at least 1 year or ascertain whether the strategy’s effect on patients persisted 1 year after diagnosis. Furthermore, the 4-month implementation period in each cluster, chosen to reduce center waiting times, may have been too short to introduce change in the wards, thus decreasing the strategy’s effect on patient outcomes. Similar issues were discussed by Turner et al^48^ as reasons for the lack of a statistically significant effect in their Promoting Optimal Outcomes in Mood Through Tailored Psychosocial Therapies Study.

A third reason for the small difference observed in our trial may be the floor effect, which arises when the effect of an intervention is measured in patients who would not need it. This problem is widely discussed in the literature as a possible cause of lack of effects of psychosocial interventions.^49,50,51^ In particular, a Cochrane review assessing the effects of psychosocial interventions on the quality of life of patients with newly diagnosed cancer^49^ did not find convincing evidence for universal implementation of individual interventions, concluding that risk screening should be conducted to target patients most in need of support. We tried preventing the potential floor effect by restricting eligibility to patients with a recent cancer diagnosis, which is a population at high risk for reduced quality of life.^49,52^ Furthermore, we investigated possible effect modifiers through subgroup analysis, and did not find effect differences according to patient level of distress, or other characteristics, such as age and sex.

A noteworthy finding of our trial is the lack of a statistically significant difference for SF. However, this domain is investigated by only 2 questions on the EORTC QLQ-C30. A general tool was preferred for this trial because it applied to patients with any cancer type.^21^ However, the use of an illness-specific instrument may have yielded a different result, as suggested by the findings of the aforementioned Cochrane review,^49^ in which a small but significant positive result was observed in the data using illness-specific measures.

Two additional elements emerging from our findings deserve consideration. First, the striking association between HQIS and the reduction of patient needs compared with usual care, although only a secondary outcome reported descriptively, suggests that the strategy may prove to be potent for helping patients cope with cancer and its treatment, with important implications for clinicians. Second, the statistically significant lower effect on EF in cluster 3 with respect to the other clusters may be due to local (cultural, organizational, and social) barriers, which should be further explored.

## Conclusions

Although our findings are positive, further research is needed. First, participation of different institution types nationwide ensures generalizability to Italian hospitals, but the effect of the HQIS in other contexts should be tested, because health care system characteristics are potential predictors of psychosocial outcomes.^45^ Second, it would be important to evaluate the effect in the longer term (3-5 years after implementation), investigating the need for strategy refreshes, as well as the longer-term effect on patient EF with a 6- and 12-month follow-up. Third, inclusion of in-depth interviews with participants (health professionals and patients) in studies of this kind may assist in defining core aspects of the intervention, which are considered beneficial, and in understanding factors underpinning acceptability.

Evidence-based psychosocial care is important for all patients. The strategy tested in this trial is feasible and has the potential to improve patient outcomes. Further research will allow refinement of the HQIS, enabling us to understand the factors that can optimize its effects.

## Notes

Supplement 1.
Trial Protocol

Supplement 2. **eMethods.** Instruments Used by the Improvement Team **eTable 1.** Percentage of Patients Who Exhibit Change in the Emotional or Social Functions of HRQoL **eTable 2.** Long-term Effect of the HQIS on the Emotional and Social Functions of HRQoL **eTable 3.** Effects of HQIS on Global HRQoL and Other Functions and Symptoms **eTable 4.** Effect of HQIS on Mood Disorder Measured With the HADS-D **eTable 5.** Results of the Subgroup Analysis Aimed to Identify Any Effect Modifiers on the Emotional Function of HRQoL

Supplement 3.
Data Sharing Statement
