# Supplementary material for: Effectiveness of a Psychosocial Care Quality Improvement Strategy to Address Quality of Life in Patients With Cancer: The HuCare2 Stepped-Wedge Cluster Randomized Trial
Source: JAMA Netw Open. 2021 Oct 14;4(10):e2128667. doi: 10.1001/jamanetworkopen.2021.28667 (PMC8517739; doi:10.1001/jamanetworkopen.2021.28667)
Supplement: Supplement 1. — Trial Protocol [file jamanetwopen-e2128667-s001.pdf]

HuCare 2 PROJECT

STUDY PROTOCOL

A stepped wedge, cluster randomized controlled trial to Assess a Strategy aiming to optimize psychosocial outcomes in patients with cancer

Promoter Associazione Italiana di Oncologia Medica

Coordinating Center UO Oncologia ASST Istituti Ospitalieri di Cremona

Sponsor No-profit

PROJECT COMMITTEES

PRINCIPAL INVESTIGATOR
Rodolfo Passalacqua (Istituti Ospitalieri Cremona - UO Oncologia)

PROTOCOL WRITING
Caterina Caminiti (Azienda Ospedaliero-Universitaria di Parma - UO Ricerca e Innovazione)
Rodolfo Passalacqua (Istituti Ospitalieri Cremona - UO Oncologia)

SCIENTIFIC COMMITTEE
Rodolfo Passalacqua (Istituti Ospitalieri Cremona - UO Oncologia)
Maria Antonietta Annunziata (IRCCS Centro di Riferimento Oncologico Aviano, SSD Psicologia Clinica)
Claudia Borreani (IRCCS Istituto Nazionale dei Tumori di Milano, SSD Psicologia Clinica)
Caterina Caminiti (Azienda Ospedaliero-Universitaria di Parma - UO Ricerca e Innovazione)
Paola Di Giulio (Università di Torino, IRCCS Istituto di Ricerche Farmacologiche Mario Negri, Milano)
Stefania Gori (Ospedale Sacro cuore Don Calabria Verona, UO Oncologia)
Carmine Pinto (IRCCS Santa Maria Nuova Reggio Emilia, UO Oncologia)
Claudio Verusio (Presidio Ospedaliero di Saronno, Varese - UO Oncologia Medica)

COORDINATION TEAM
Barbara Marcomini: quality assurance assistant (Azienda Ospedaliero-Universitaria di Parma, UO Ricerca e Innovazione)
Elisa Iezzi: data-manager & statistician (Azienda Ospedaliero-Universitaria di Parma, UO Ricerca e Innovazione)
Silvia Lazzarelli: senior clinical research assistant (Istituti Ospitalieri Cremona, UO Oncologia)
Jessica Saleri: psicologa esperta analisi del contesto (Istituti Ospitalieri Cremona, UO Oncologia)
Sabrina Zora: psicologa project manager (Istituti Ospitalieri di Cremona, UO Oncologia Medica)
Monica Cattaneo: infermiere di ricerca per supporto ai centri (Istituti Ospitalieri Cremona, UO Oncologia Medica)
Raffaele Maddalena: coordinatore infermieristico per supporto ai centri (AO di Desio e Vimercate, MB Dipartimento di Oncologia)
Vito Donati: coordinatore infermieristico per supporto ai centri (Istituti Ospitalieri Cremona, UO Oncologia Medica)
Pietro Zerla: coordinatore infermieristico per supporto ai centri

SCIENTIFIC-ORGANIZATIONAL SECRETARY
Francesca Diodati, UO Ricerca e Innovazione, Azienda Ospedaliero-Universitaria di Parma
Tel: 0521 703697 **Fax:** 0521 703925 **e-mail:** fdiodati@o.pr.it
Elisa Colucci, UO Oncologia, Istituti Ospitalieri di Cremona **Telefono:** 0372 405237 **Fax:** 0372 408214
e-mail: segreteria.oncologia@asst.cremona.it

AIOM Servizi
Tel: 02 26683129 **Fax:** 02 596105559 **e-mail:** info@aiomservizi.it

SYNOPSIS

**TITLE**	A stepped wedge, cluster randomized controlled trial to Assess a Strategy aiming to optimize psychosocial outcomes in patients with cancer
**STUDY DURATION**	30 months (expected study initiation date May 2016 – expected study completion date October 2018)
**NUMBER OF CENTERS**	15 cancer centers located nationwide, grouped into 3 clusters according to geographical location
**RATIONALE**	Despite guidelines recommending psychosocial care interventions in oncology, many patients who would benefit from such interventions actually do not receive them.<br>This issue has inspired an implementation study (HuCare), conducted in 28 Italian cancer centers, which demonstrated the feasibility of a strategy, HuCare Quality Improvement Strategy (HQIS), aimed to integrate into practice 6 psychosocial interventions recommended by international guidelines.<br>Starting from the positive results of the HuCare study, this trial intends to assess whether HQIS introduction improves quality of life of cancer patients.
**OBJECTIVES**	Primary objective is to evaluate effectiveness of HQIS vs standard care, in terms of improvement of at least one of two functional domains (emotional or social) of Health-Related Quality of Life (HRQoL), detected at baseline (before treatment initiation) and at clinical follow-up (after about 3 months). Primary endpoint will be measured with the EORTC QLQ-C30 questionnaire, version 3.0 validated into Italian.<br>Secondary objectives are:<br>a) To investigate whether the strategy's effect lasts through time (long-term effect), impacts on specific patient categories, modifies global HRQOL or only given domains, reduces mood disorders<br>b) To measure implementation rate in participating centers, in terms of percentage of clinical staff members (medical oncologists and nurses) who complete training, percentage of patients with unmet psychosocial needs and percentage of patients who systematically receive the strategy
**STUDY DESIGN**	multicenter, Stepped-Wedge, Cluster Randomized Trial (SWD-CRT), where the strategy is carried out sequentially in three clusters (each comprising 5 centers) and in three equally spaced epochs. Implementation sequence will be randomly determined and by the end of the trial all centers will have implemented the HQIS strategy. The intervention is applied at a cluster level, and assessed at an individual level (on patients).
**STRATEGY**	The HQIS lasts 16 weeks (4 months) and comprises three phases:<br>**Health care professional training**<br>Medical and nursing staff of participating centers attends a training course (recommendation 1) to improve communication-relational skills<br>**Center support**<br>The Improvement Team (IT) made up of external personnel (sociologist,

	psychologist and research nurse), conducts 4 on-site visits at the centers (Start-up, Support, Implementation and compliance)<br>**Implementation of recommendations**<br>Besides clinician training, 5 additional recommendations identified in the HuCare Project will be implemented in each cluster:<br>After the first visit and before treatment initiation, the oncologist proposes to the patient the use of a question prompt list (recommendation 2), to facilitate communication, and is assigned a specialist nurse (recommendation 3).<br>Each patient is screened for psychological distress (recommendation 4) and social needs (recommendation 5) and appropriate counselling and/or services are activated as necessary (psychologist, psychiatrist, social worker, etc.).<br>Before treatment is initiated, patients are offered a meeting with the nurse (recommendation 6) at the Point of Information and Support (PIS), a room dedicated to consultations with patients.
**INCLUSION CRITERIA**	Patients with cancer, of any type and stage, who consecutively access participating centers in an index period and who satisfy the following eligibility criteria:<br>o Age $\geq$ 18 years<br>o Diagnosis (histological or cytological) of solid cancer notified to the patient within two months<br>o Who are about to start a new medical treatment: chemotherapy (both I.v. and oral), molecular target drugs, hormonal therapy, immunotherapy.<br>o Expected survival > 3 months<br>o Good comprehension of the Italian language<br>o Who have read, understood, and signed the informed consent
**EXCLUSION CRITERIA**	o Previous chemotherapy or other medical cancer treatment<br>o Enrolment in the same trial in a previous epoch<br>o Simultaneous participation to other trials which entail self-administration of questionnaires/scales measuring anxiety, depression or quality of life (Patients Reported Outcomes, PROs)<br>o Hospitalized<br>o Treated for psychiatric disorders<br>o Affected by mental or psychiatric disorders, due to cancer or coexisting illness, which interfere with awareness or judgemnt ability<br>o Inability to complete the questionnaire or to comply with 3-month follow-up
**SAMPLE SIZE**	The number of subjects to be enrolled was defined following the methodology for incomplete, cross-sectional Stepped-wedge cluster randomized trials, assuming a generalized linear mixed model. Considering an expected difference deemed clinically acceptable between 3 and 8 points of at least one functional domain (social or emotional), an ICC (Intra-class correlation coefficient) of 0.80 and a 20% drop-out at FU, the

	overall number of subjects to enroll is 720 subjects , i.e. 60 patients for each cluster at each of the 4 epochs.
**STATISTICAL ANALYSIS**	Differences of HRQoL values between the two groups, post-intervention and control, for each of the two functional domains (emotional and social) of interest for the primary objective, will be analyzed using a binomial Beta regression model (BB, due to the asymmetric value distribution. This model also enables to estimate the strategy's effect in terms of Odds Ratio.
**FEASIBILITY STUDY**	Before trial initiation, a pilot study will be performed at the cancer center of Cremona, on a consecutive sample of patients accessing the ward over two weeks. The aim is to measure feasibility and acceptability of questionnaire administration using a tablet.

ABBREVIATIONS

**AIOM**	Associazione Italiana di Oncologia Medica – Italian Association of Medical Oncology
**EBM**	Evidence Based Medicine
**EC**	Ethics Committee
**CONSORT**	Consolidated Standards of Reporting Trials
**EP**	End Point
**eCRF**	Electronic Case Report Form
**EORTC QLQ-C30**	European Organization for Research and Treatment of Cancer Quality of Life Questionnaire-Core 30 items (
**HADS**	Hospital Anxiety and Depression Scale
**HQIS**	HuCare Quality Improvement Strategy
**HRQoL**	Health-Related Quality of Life
**HuCare**	Humanization in Cancer Care
**IQR**	interquartile range (cioè 25° e 75° quartile),
**IT**	Improvement Team
**ITT**	Intention-To-Treat (population)
**PP**	Per-Protocol (population)
**PRO**	Patient-Reported Outcome
**RCT**	Randomized Clinical Trial
**RECIST**	Response Evaluation Criteria in Solid Tumors (linea guida per la valutazione della risposta tumorale)
**NEQ**	Needs Evaluation Questionnaire (NEQ)
**SC**	Scientific Committee
**SD**	Standard Deviation
**SWD-CRT**	Stepped-Wedge Design Cluster Randomised Trials

1 INTRODUCTION

1.1 BACKGROUND

Cancer has a significant impact on the lives of patients and their families, which is not restricted to symptoms and treatment side effects. Research points to the existence of a wide range of psychosocial needs, defined as psychological, emotional, social and spiritual aspects of health, which frequently are not detected or adequately dealt with [Howell 2012]. Although prevalence of psychological distress among cancer patients is difficult to estimate, because of the many different tools and diagnostic criteria used, research reports that up to 75% of newly diagnosed cancer patients develops psychological distress [Galway 2012]. Furthermore, the burden of the disease is increased by frequent practical demands (economic problems due to absence from work, transportation to reach the hospital, etc.) and information needs concerning the disease and available support services [Harrison 2009, Howell 2012]. These aspects are so relevant that Bultz and Carlson suggest to consider cancer a Bbiopsychosocial illness[Bultz 2006] and to strongly advocate for the need to integrate the psychosocial domain into practice [Bultz 2006, Holland 2011].

Health-related quality of life (HRQoL) is a multidimensional concept referring to the effect of an illness and its therapy upon a patient's physical, psychological and social wellbeing as perceived by the patient himself [Roila 2001]. Numerous studies, conducted in different countries, show that HRQoL of patients affected with diverse types of cancers is lower than that of the general population [Kenzik 2015, Choi 2015, Aro 2015], and that the risk of a reduced QoL is greater at certain stages of the care process, such as the first few months of treatment, a particularly critical period characterized by high levels of anxiety and depression [Aro 2015, Stanton 2006].

Today there is a general agreement on the importance of HRQoL as an outcome in clinical trials [Basch 2012]. As back as 1996, the American Society of Clinical Oncology recommended that it be considered a primary outcome in any phase III trial [American Society of Clinical Oncology 1996]. Similarly, the Food and Drug Administration supports the importance of incorporating Patient-Reported Outcomes (PRO), such as QoL, both in cancer research, and in the decision process for the approval of medicinal products in oncology [Johnson 2003]. A review of phase III studies performed by the National Cancer Institute of Canada Clinical Trials Group shows how detection of HRQoL, not only represents an added value in research, but in some cases can also even modify the clinical interpretation of trial results [Au 2010].

HRQoL also plays a central role as a measure of the quality of care [Halyard 2008, Bultz 2015]. The policy statement of the European Partnership Action Against Cancer Consensus Group, an initiative of the European Commission launched in 2009, considers the quality of life of a patient an essential element in the decision-making process, which must be discussed with the patient [European Partnership Action Against Cancer consensus group 2014].

Given the relevance of HRQoL for people with cancer, the literature is increasingly emphasizing the importance of identifying ways of maintaining and improving their quality of life [Galway]. In this regard, a Cochrane systematic review [Galway 2012] summarizes findings from 30 randomized and quasi-experimental trials (5.155 patients, of 1249 patients included in the meta-analysis), published between 1981 and 2009, aimed at assessing the effect in terms of HRQoL (as the primary outcome) of a psychosocial intervention comprising an interpersonal relationship between patients and specifically trained health care professionals. The authors conclude that no statistically significant results have been obtained on HRQoL at 6 months, probably also due to methodological limitations, heterogeneity of the detection tools used, high risk of contamination bias and of a dilution of the observed effect. Based on these considerations, the review provides indications for future research, emphasizing the need for randomized controlled trials on those patients who are more likely to benefit from psychosocial interventions, and using adequate tools and sensitive measures, i.e. capable of capturing changes in the psychosocial domain.

1.2 Rationale

Although various guidelines exist providing evidence-based recommendations on psychosocial care in cancer [Jacobsen 2012], evidence suggests that many patients with cancer who might benefit from these interventions do not receive them [Jacobsen 2015]. This fact formed the basis for an implementation study (HuCare), funded by the Ministry of Health and by the Lombardy Regional Health Trust, conducted in 28 cancer centers nation-wide, completed in 2014. This project evaluated the feasibility of a strategy (the "HuCare Quality Improvement Strategy - HQIS), aimed at integrating into practice 6 psychosocial interventions, recommended by international guidelines. The strategy, based on context analysis and problem solving, includes communication skills training for clinical staff and the support by an external team, to assist staff in identifying obstacles, finding solutions, and strengthening

motivation to carry out recommended changes. HuCare has demonstrated the strategy's feasibility in a real context [Passalacqua 2016], since over 75% of patients had received the psychosocial interventions in 27 of the 28 participating centers.
Building on the findings of HuCare, this randomized trial was therefore designed to
Assess whether the introduction of the HQIS in oncology wards improves the quality of life of cancer patients. This trial is based on the following hypotheses.
- improving communication and relational skills of clinical staff (medical oncologists and nurses) facilitates change of behavior
- providing support for context analysis and for the solution of problems detected at a local level favors implementation of recommended psychosocial interventions
- carrying out such recommendations improves quality of life of cancer patients who initiate active treatment, i.e. who are at high risk of experiencing a decrease in their HRQoL.

The study follows the methodology outlined in the Medical Research Council guidelines on complex interventions [Craig 2008], in the CONSORT Statement and its extensions (Patients Reported Outcomes (PRO) Extension and Extension to Cluster Randomised Trials) [www.equator-network.org].

2. Objectives and ENDPOINTS

2.1 Primary objective

The primary objective is to assess the effectiveness of the HQIS vs standard care, in terms of improvement of at least one of the two functional domains of HRQoL, emotional or social, detected at baseline (before treatment initiation) and at clinical follow-up (approximately 3 months after study enrolment).
The primary endpoint will be measured using the EORTC QLQ-C30 questionnaire (see Paragraph 8.1), version 3.0 validated into Italian.

2.2 Secondary objectives

a) To investigate whether the strategy has an effect:
- on patient mood
- in the long-term
- on overall HRQoL or on specific domains
- on specific patient types (case-mix)
b) To measure implementation rates, in terms of:
- percentage of clinical staff who complete training (see paragraph 5)
- percentage of eligible patients who systematically receive the strategy

3. STUDY DESIGN

This is a multicenter, incomplete stepped-wedge cluster randomized controlled trial (SWD-CRT) (since data are not collected during implementation), where the intervention strategy is sequentially carried out in three groups of centers (clusters with 5 centers each) and in three equally spaced periods of time (every 4 months, from 2nd to 4th epoch). The study also includes an initial epoch during which none of the centers is exposed to the intervention, and a final epoch when all centers will have implemented the strategy [Hemming 2015, BMJ; Hemming 2015, Stat Med]. The implementation epoch for each center is randomly assigned, and by the end of the study, all centers will have received the strategy. The intervention is applied at a cluster level, which constitutes the unit of randomization, and assessed at an individual level, (on the patients of each cluster) with cross-sectional model (for each period, patients are different).

	Centers	Epoch 1 (Months 1-4)	Epoch 2 (Months 5-8)	Epoch 3 (Months 9-12)	Epoch 4 (Months 13-16)	Epoch 5 (Months 17-20)
Cluster 1	1	Control	HQIS Implementation	Post-Intervention	Post-Intervention	Post-Intervention
	2	Control	HQIS Implementation	Post-Intervention	Post-Intervention	Post-Intervention
	3	Control	HQIS Implementation	Post-Intervention	Post-Intervention	Post-Intervention
	4	Control	HQIS Implementation	Post-Intervention	Post-Intervention	Post-Intervention
	5	Control	HQIS Implementation	Post-Intervention	Post-Intervention	Post-Intervention
Cluster 2	6	Control	Control	HQIS Implementation	Post-Intervention	Post-Intervention
	7	Control	Control	HQIS Implementation	Post-Intervention	Post-Intervention
	8	Control	Control	HQIS Implementation	Post-Intervention	Post-Intervention
	9	Control	Control	HQIS Implementation	Post-Intervention	Post-Intervention
	10	Control	Control	HQIS Implementation	Post-Intervention	Post-Intervention
Cluster 3	11	Control	Control	Control	HQIS Implementation	Post-Intervention
	12	Control	Control	Control	HQIS Implementation	Post-Intervention
	13	Control	Control	Control	HQIS Implementation	Post-Intervention
	14	Control	Control	Control	HQIS Implementation	Post-Intervention
	15	Control	Control	Control	HQIS Implementation	Post-Intervention

Figure 1 – Study design: stepped wedge cluster randomized controlled trial

The cluster randomized design was selected because the intervention is organizational and requires high involvement of all center staff; therefore, randomizing individual oncologists or patients would not be possible, as it would entail a high risk of contamination bias. Furthermore, the stepped-wedge design enables to overcome the logistic difficulty of simultaneously providing the intervention to all centers, and it is ethically acceptable, since it ensures that all patients may receive an intervention considered to be beneficial. Finally, the SWD-CRT offers the opportunity to measure the effect of the intervention in time (secondary objective), which is one of the gaps highlighted in the literature [Ament 2014, Passalacqua 2016].

4. STUDY POPULATION

4.1 Setting

The project was presented in October 2015 at the national conference of the Italian Association of Medical Oncology (AIOM), during a meeting open to interested members. Study recruitment was performed using the "Facilities Questionnaire", a brief survey developed for the project to ascertain the presence of essential prerequisites for study conduction (Attachment I), and to ensure representativeness of centers according to size and geographical location. The number of centers to be included has been determined based on feasibility (costs and logistic restrictions) and clusters defined geographically (North, Center-South, Islands).
The Directors of participating wards declared that all staff was informed on the aims and conduction of the project and accepted to take part in the study.

4.2 Study patients

Cancer outpatients of any type and stage, who consecutively access the participating centers during an index period and who fulfill the following eligibility criteria:

Inclusion criteria
Patients with cancer, of any type and stage, who consecutively access participating centers in an index period and who satisfy the following eligibility criteria:

- Age $\geq$ 18 years
- Diagnosis (histological or cytological) of solid cancer notified to the patient within two months
- Who are about to start a new medical treatment: chemotherapy (both I.v. and oral), molecular target drugs, hormonal therapy, immunotherapy.
- Expected survival > 3 months
- Good comprehension of the Italian language
- Who have read, understood, and signed the informed consent

Exclusion criteria

- Previous chemotherapy or other medical cancer treatment
- Enrolment in the same trial in a previous epoch
- Simultaneous participation to other trials which entail self-administration of questionnaires/scales measuring anxiety, depression or quality of life (Patients Reported Outcomes, PROs)
- Hospitalized
- Treated for psychiatric disorders
- Affected by mental or psychiatric disorders, due to cancer or coexisting illness, which interfere with awareness or judgemnt ability

Inability to complete the questionnaire or to comply with 3-month follow-up

5. STRATEGY

The HuCare Quality Improvement Strategy takes 16 weeks to complete (4 months); Figure 2 outlines its three phases:

a) Clinician training

The medical and nursing staff of participating centers will attend an Evidence-Based Medicine (EBM) course (recommendation 1) [Fallowfield 2003, Brown 2008, McLeod 2014] to improve communication-relational skills and to receive instructions on how to enact the recommendations of the project (Attachment II). Considering an average of 8 oncologists and 14 nurses for each center (based on the HuCare study experience), 2 editions of the course for oncologists and 3 editions for nurses will be provided for each cluster group, during a period of 6 consecutive weeks. All editions will be held on weekends in Milan, at the AIOM HuCare School for Communication in Oncology located at Palazzo delle Stelline. Each center must ensure that at least 75% of medical staff and nursing staff operating in the ward complete training.

b) Center support

Centers will be provided with a reference manual with useful instructions for rapid implementation of the interventions in their context. The Improvement Team (IT) composed of personnel not employed at the center (sociologist MGO, psychologist JS and nurse coordinators GD/RM/PZ), will perform 4 on site visits over a 10 week period (Start-up, Support, implementation and compliance visits). The main purpose of each visit are summarized in Figure 2.

c) Implementation of recommendations

Together with training (recommendation 1), 5 more recommendations for the psychosocial care of patients, identified in the HuCare project, will be implemented at each center by trained staff and with the support of the IT.

After the first visit with the oncologist, and before initiation of treatment, all "new" patients will receive the following activities, aimed to satisfy their information needs and to facilitate communication with the oncologist:

- The oncologist encourages the patient to use a question prompt list (recommendation 2), to favor communication. The tool was created in the framework of the HuCare project, by cross-cultural adaptation [Caminiti 2010] (Attachment III). The oncologist also introduces to each patient his or her specialist nurse (recommendation 3), who will accompany the patient through his/her care.
- The specialist nurse provides the patient with the two self-administered questionnaires, HADS and NEQ, validated into Italian (see paragraph 8), to detect psychological distress (recommendation 4) and social needs (recommendation 5). For patients with distress and/or social needs, necessary counselling and/or services (psychologist, psychiatrist, social worker, etc.) are activated, according to a written procedure defined at each center before the implementation phase begins. The specialist nurse also offers the patient a meeting at the Point of Information and Support (PIS), a space reserved for patients, equipped with internet access and information material. The PIS should be adjacent to the oncology offices, in a location ensuring privacy (recommendation 6).

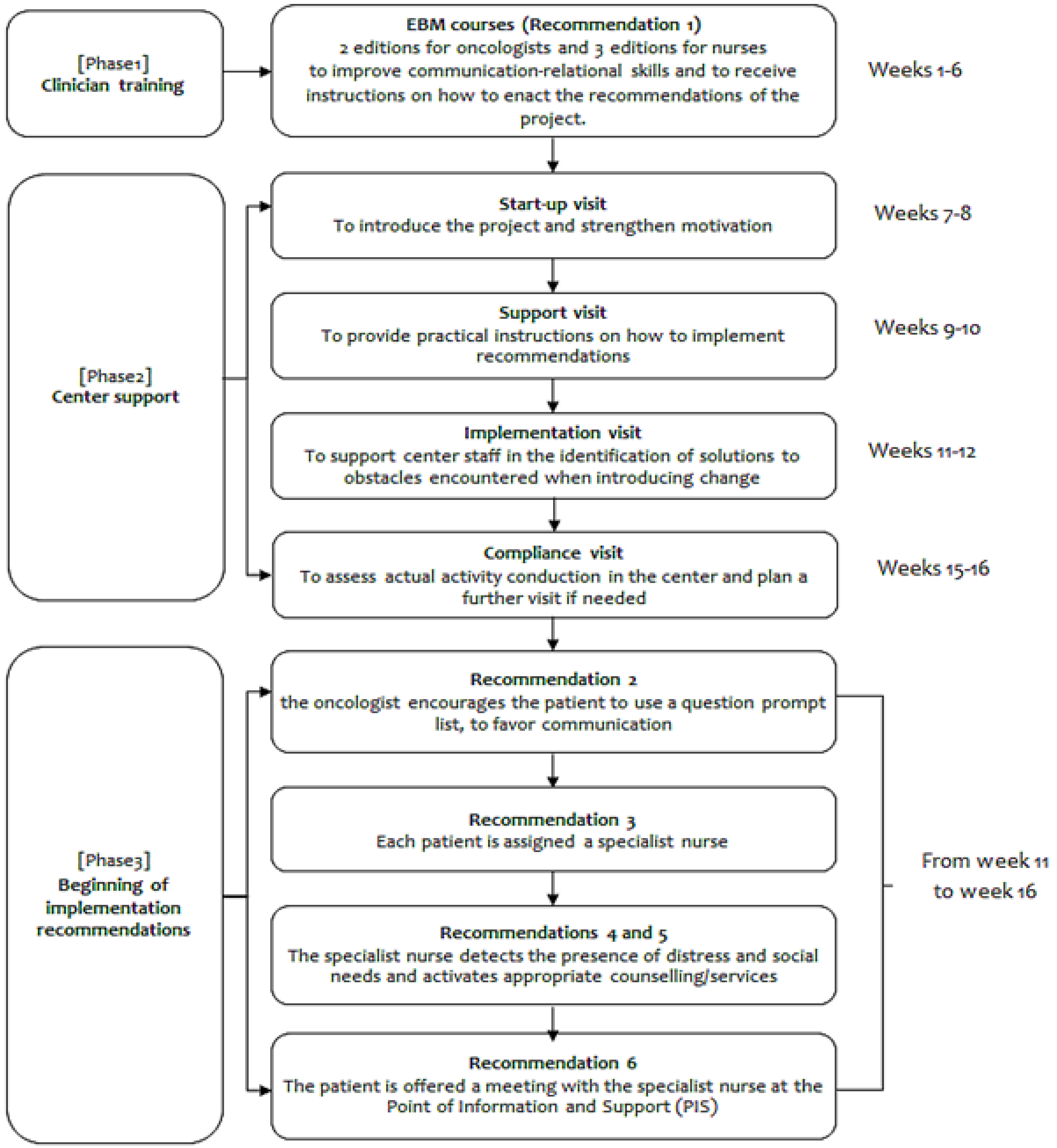

Figure 2 Hucare Quality Improvement Strategy (HQIS) Flow chart

6. OUTCOMES

The study's primary end point is the difference between the means of changes of individual scores detected at baseline and at follow-up (within each group), during the post-intervention period compared with control periods (between groups).

The following secondary outcomes will also be assessed, again comparing baseline values with

measurements at 3 month follow-up up, during the post-intervention periods vs control periods, in order to test HQIS impact:

- in the long-term, i.e. whether different effects on patients initiating new treatment are detected over one year
- on global HRQoL, on specific scales and on individual symptoms, detected by the EORTC QLQ-C30 questionnaire
- on mood disorders, measured with the HADS-D scale
- according to patient subtypes with different baseline values of anxiety and depression [Zhu 2016].

To assess the actual degree of strategy's implementation at individual centers, the following secondary outcomes will be measured:
- percentage of clinical staff (oncologists and nurses) who complete training (see paragraph 5)
- percentage of patients with unmet social needs, detected with the NEQ (Needs Evaluation Questionnaire(see paragraph 8.1)) at baseline vs three-month follow-up
- percentage of eligible patients who receive HuCare recommendations, recorded during the implementation visit (csee paragraph 5 and Figure 2) on a sample of 5 consecutive cases for each center, by reviewing patients' clinical records where performed psychosocial interventions must have been noted.

7. TIMING

Patients will be screened and enrolled over 2 consecutive index weeks, to ensure necessary sample size and representativeness of different cancer types and treatments administered at the centers. Data collection will take place at two distinct time points:

- enrolment phase, when baseline patient data will be recorded
- assessment phase, when data for the same patients at 3-month follow-up will be recorded

Timing	**Epoch 1**<br>From month 1 to 4	**Epoch 2**<br>From month 5 to 8	**Epoch 3**<br>From month 9 to 12	**Epoch 4**<br>From month 13 to 16	**Epoch 5**<br>From momth 17 to 20
Enrolment					
Screening, Informed consent and Baseline questionnaire Cluster 1	X		X	X	X
Screening, Informed consent and Baseline questionnaire Cluster 2	X	X		X	X
Screening, Informed consent and Baseline questionnaire Cluster 3	X	X	X		X
Intervention					
HQIS Implementation Cluster 1	Control	Implementation	Post-Intervention	Post-Intervention	Post-Intervention
HQIS Implementation Cluster 2	Control	Control	Implementation	Post-Intervention	Post-Intervention
HQIS Implementation Cluster 3	Control	Control	Control	Implementation	Post-Intervention
Assessment					
Questionnaires at 3-month follow-Up Cluster 1	X		X	X	X
Questionnaires at 3-month follow-Up Cluster 2	X	X		X	X
Questionnaires at 3-month follow-Up Cluster 3	X	X	X		X

Table 1 –Data collection schedule

Effect measure at three months was chosen because QoL trend was observed to reach its negative peak 3 months after enrolment, and then to improve over one year [Aro 2015]. This trend may be explain by the adjustment trajectory of patients, who after an initial traumatic phase are able to adapt to the illness [Galway 2012]. Cocks et al. [Cocks 2012] also indicate 3 months as the ideal timing to use in studies measuring intervention impact on HRQoL, since it increases study efficiency (smaller sample size) given the wider difference between baseline and 3- months follow-up scores.

7.1 Eligibility assessment

After the first consultation with the oncologist and before initiation of the first cycle of therapy, outpatients consecutively accessing the center will be screened by the oncologist over a period of two index weeks, indicated by the statistician. To this end, the oncologist checks the presence of all inclusion criteria and absence of all exclusion criteria, introduces the study to eligible patients, provides the information sheet and collects the informed consent to participation before initiation of the first cycle of therapy.

7.2 Baseline assessment

Prior to initiation of the first cycle of therapy, and before implementing the recommended interventions addressed to patients, a specially trained research nurse of the center will enter demographic and clinical variables (taken from medical records) into the electronic data collection form (eCRF), and will instruct the patients on how to complete the questionnaires using a tablet. The time needed for completion is estimated not to exceed 20 minutes for each patient (12 minutes for the EORTC and 8 minutes for the other questionnaires). The research nurse will check completeness of entered information, and in the case of missing data will invite the patient to fill in the empty fields, noting any problems arisen in the process.

7.3 Three-month Assessment

Before the medical follow-up appointment, the research nurse will invite the patient to complete the questionnaires using the tablet. At the end of the visit, the nurse will enter the following information taken from the clinical record into the eCRF: ECOG at 3 months, any disease progression (according to RECIST criteria), and any reasons for incomplete questionnaires and for the premature exit from the study (lost to FU, deceased, withdrawal).

8. STATISTICAL METHODS

Data will be processed with SAS software Version 8.2 (Statistical Analysis System) and STATA/SE version 11.0.
Before database lock and cleaning, the Statistical Analysis Plan (SAP) will be defined, comprising the following essential elements:

- Measure calculation and interpretation
- Statistical methods for the analysis
- Management of missing data
- Tables, lists and figures for data collection monitoring and for the final analysis.

8.1 INSTRUMENTS FOR OUTCOME MEASUREMENT

EORTC QLQ-C30

Quality of life, primary endpoint of the study, will be assessed with the validated Italian version of the European Organisation for Research and Treatment of Cancer Quality of Life Questionnaire-Core 30 items (EORTC QLQ-C30) [Apolone 1998], specific for patients with cancer . The tool is self-administered, and comprises 30 questions, 24 of which form nine multi-item scales representing the different aspects, or domains, of QoL: a global health status / QoL scale, five functional scales (physical, role, emotional, cognitive and social), and three symptom scales (fatigue, pain, nausea and vomiting), as well as 6 single items assessing additional symptoms (dyspnoea, loss of appetite, insomnia, constipation, diarrhea) and perceived financial impact of the disease).
As required in the Consort PRO extension [Calvert 2013], we briefly describe the ways the instrument and relative parameters for the assessment of the main endpoint were selected.
Various tools validated in many languages exist in the literature for the measurement of HRQoL, but two of them are most commonly used: the EORTC QLQ-C30 [Osoba 1994] and the FACT-G [Cella 1993] (Functional Assessment of Cancer Therapy-General). Luckett et al. [Luckett 2011] report an interesting comparison between the two tools, concerning content, scale, structure, psychometric properties, and other aspects, and constructed an algorithm which helps researchers in choosing the more adequate questionnaire according to the items of interest. Another recent work [King MT 2014] compares the two instruments' "responsiveness", or sensitivity to change, the most important property of a questionnaire used within a trial aiming to demonstrate the impact of an intervention, defined as the ability of an instrument to detect the minimal change considered to be clinically important by patients [Uwer 2011], and statistical efficiency, i.e. the sample size needed to detect such effects. Considering the nature of psychosocial interventions implemented in this study, which we hypothesize to mainly impact the social and emotional domains, and following the indications of the aforementioned papers, the QLQ-C30 was selected.
The choice of the three domains and corresponding timing, i.time point of effect measurement, was performed based on the analysis of the work by Cocks et al [Cocks 2012] with the aim to restrict assessment to the emotional and social functions which are mostly affected by the psychosocial intervention and to the population at greatest risk of QoL deterioration (patients during the first 3 months of treatment).
The questionnaire is self-administered using a touch-screen tablet device, at baseline and three months after enrolment, during the follow-up visit. The electronic version was chosen as it reduces completion times [Pollom 2015; Kesterke 2015] and the risk of missing data and entry errors [Marcano Belisario 2015], making QoL detection more efficient and accurate [Berry 2015]. Furthermore, the electronic

device for PRO detection has been shown to be easily used also by people over 70 years old, who only require a few more minutes than the younger population [Pollom 2015; Kesterke 2015].
To ensure the correct use of the questionnaire during data collection, as well as appropriate data analysis and interpretation, the indication of the EORTC manual will be followed.

Hospital Anxiety and Depression Scale

The HADS is a self-report questionnaire, validated into Italian [Costantini 1999], comprising 14 items, 7 assessing the level of anxiety (HADS-A) and 7 assessing the level of depression (HADS-D), with scores for each item ranging from 0 to 3 and an overall score range between 0 and 42. For each statement, patients are asked to select among four options the one which best describes his/her emotional state referred to the previous week.

Needs Evaluation Questionnaire

Social needs are detected using the NEQ, a tool developed in Italy, composed of 25 items, intended to record the main, potentially manageable social needs connected to the state of health, of patients with cancer [Tamburini 2000]. Identified areas concern information, communication and relationship with health care professionals; symptoms or functional difficulties; involvement of other professionals (social worker, psychologist, spiritual advisor); financial issues and help with lodging; psychological needs at an individual, family and social level.

8.2 Variable coding

The following main patient demographic and clinical characteristics are collected at baseline to describe the study population and determine factors associated with quality of life:

- Date of birth (dd/mm/yyyy)
- Gender (M/F)
- Civil status (married or living with partner, other)
- Education (primary, high-school and above)
- Date of diagnosis (dd/mm/yyyy)
- Presence of metastases (yes/no)
- Cancer site
- ECOG performance status
- Type of treatment (IV chemotherapy , oral drugs, molecular target drugs)

At three-month follow-up, presence of disease progression will also be recorded (yes/no). For questionnaires where information was missing (less than half of items was completed), reasons for non-completion will also be noted, i.e. patient withdrawal, patient refusal to complete the item, worsening of patient's health conditions hindering completion, patient lost to follow-up or deceased (see paragraph 8).

As for coding of QLQ-C30 questionnaire variables, responses are given on likert scales and the sum of assigned values yields the score for each of the 15 domains. Highest values in the symptom domain (e.g. Diarrhea, weakness, vomiting etc.) will indicate severe symptomatology. Highest values in the other domains will indicate better quality of life. Scores of each domain are linearly transformed into a 0-100 scale, where 0 and 100 are assigned to the lowest and highest possible value, respectively.
For the HADS-D scale [Annunziata 2011] a cut-off of >7 will be used, a score considered clinically significant (i.e. patients scoring above this value may benefit from psychological support therapy) and with high sensitivity (0.86) and specificity (0.81) [Vodermaier 2011]. Missing values will be replaced with

the mean of the available values for anxiety or depression, if not more than four values are absent.
The NEQ detects the presence of different types of needs using dichotomic choices (yes/no). It will enable to assess the frequency of needs and to compare it across the two detection periods (baseline and follow-up). Specifically, this information will allow to determine whether the HQIS is effective in reducing needs and whether this also corresponds to a positive effect on quality of life.

8.3 Data management

Data will be gathered anonymously by means of an eCRF which uses a remote single-entry system with electronic check of data congruence. Together with the usual data entry functions and on-line checks, this system, employing mobile devices (tablet, smartphone), created at the Research and Innovation Unit of Parma, also includes a control check of operator identification (investigator), check of patient eligibility criteria and of data entry required by the three questionnaires used in the study. To reduce missing data, a banner will appear warning the patient to complete all fields or to indicate unwillingness to answer, when some fields are left empty. If > 50% of responses is missing, the research nurse will indicate the reasons in the eCRF (see paragraph 8.9).

8.4 Randomization

The unit of randomization is the cluster, a group of 5 centers randomly assigned to one of the HQIS implementation epochs; given 15 participating centers, 3 clusters will be formed, each including 5 centers located in the same geographical area (North, Center, South-Islands) to facilitate the work of the IT. The sequence of strategy implementation is defined by the statistician (EI) through SAS software, who will inform centers of their assigned implementation epoch with a 4-week notice. The unit of statistical analysis, on which the primary and secondary endpoints (excluding the percentage of clinical staff completing training), are patients enrolled by clusters during two index weeks, randomly selected by the statistician.

8.5 Blinding

Blinding will be ensured both for patients, who will not be aware of the study epoch (control period or post-intervention period)in which they are providing information on their HRQoL, and for the statistician, who will use anonymized data and crypted identification codes for the study epochs. This should prevent ascertainment, performance, and attrition biases [Spirit guideline, www.equator-network.org]. The nature of the intervention precludes blinding for clinical staff.

8.6 Sample Size

The number of subjects to be enrolled was defined following the methodology for incomplete, cross-sectional Stepped-wedge cluster randomized trials [Hussey 2007, Hemming 2013, Hemming 2014; Hemming 2016] considering:

a) 3 clusters, each comprising 5 centers and with equal size for each cluster (capacity of enrolment per week);
b) an expected mean difference deemed clinically acceptable lying between 3 and 8 points of at least one domain (social or emotional), values indicated in the paper by Cocks et al [Cocks 2011], as minimal clinically relevant differences for the domains of this study;

c) an ICC (Intra-class correlation coefficient) equal to 0.80, as reported in two papers [Uwer 2011, Lundy 2014];
d) the Wald Test, with time as fixed effect and cluster as the random effect [Hussey 2007]
e) a power of 80% and two-tail alpha of 5%;
f) drop-out of 20% at follow-up [Ledderer 2013].

Applying the *steppedwedge* di STATA/MP 11.2 procedure [Hemming 2014] we calculated an overall sample size of 720 patients, which means 60 patients in each cluster for every detection period.

	t1	t2	t3	t4	t5
c3= 5 unit	60	60	60		60
c2=5 unit	60	60		60	60
c1 =5 unit	60		60	60	60
	180	120	120	120	180

8.7 Statistical analysis

Sample descriptive characteristics will be presented as means and standard deviation (SD) when normally distributed, or as medians and interquartile ranges (IQR). Although the majority of responses to individual items/symptom/functional scale of the EORTC QLQ-C30 questionnaire exhibits asymmetric distribution, findings will nevertheless be described with both mean and median values.
The main unit of analysis is the individual. Differences of HRQoL values between the two groups, post-intervention and control, relative to each of the two functional domains (emotional or social) of interest for the primary objective, will be analyzed using a binomial Beta regression model (BB), as suggested by different authors [Khan 2015, Arostegui 2012], due to the asymmetric distribution of values. This model also enables to estimate the strategy's effect in terms of Odds Ratio, the preferred measure by oncologists for its more immediate interpretation and greater usefulness in clinical practice, compared with HRQoL absolute values [Khan 2015]. To carry out the analysis with the BB model, responses will be transformed into a scale (0,1) by using the formula Y-a / b-a, where a and b are the lowest and highest possible scores, respectively, and Y is the observed response. For instance, a score of 80 will be expressed as 80-0/(100-0) = 80/100 =0.8.
Since the study is an incomplete, cross-sectional stepped-wedge cluster randomized controlled trial (with an implementation period) the following covariates will be included in the model: the implementation epoch (first, second or third) and time of exposure to the strategy; the cluster the patient belongs to (1, 2 or 3) and intra-cluster correlation [Hemming 2015, Stat Med].
The demographic and clinical variables which influence the outcome with a *p value<0.20* in the univariate analysis will be included in the regression model. Data analysis will be conducted using SAS v 8.1 and STATA/SE 11.2

8.8 Analyzed population

For the principal analysis of effectiveness, an Intention-to-treat population (ITT) is considered, composed of all eligible patients with HRQoL assessments at baseline and at 3-month follow-up. Patients are considered to be exposed to the intervention according to randomization, regardless of

any delay or failure to conduct the intervention. A “Per-protocol population” (PP) will also be assessed, composed of centers which will complete the trial without any breaches to the protocol and which exhibit a degree of compliance (% of trained clinicians and % of eligible patients who have received the intervention) greater than 75%. Sensitivity analyses will be conducted to assess the robustness of the missing data assumption made in the primary analysis.

8.9 Missing data

Data will be classified in two ways, according to the degree of completion: missing responses (questionnaires with one or more missing responses) or missing questionnaire (questionnaires with more than half missing responses). These two cases will be managed differently in the analysis process, as described below:

1. *Missing responses*
   The expected proportion is 2%, as reported in the EORTC QLQ-C30 Scoring Manual,[66] with missing values casually distributed in the two groups. To reduce the proportion of missing data, patients who have not filled in all fields at the end of completion will see a banner reminding them to do so, or to state unwillingness to respond. During data analysis, if a response is given for at least half of the items, the missing values are assumed to correspond to the mean of the given responses and the imputation method deemed most appropriate will be applied. If the number of missing data is greater than the half of the items, the questionnaire will not be considered valid.[66]

2. ***Missing questionnaire***
   If no response is given, or if the number of missing responses exceeds half of the items, the research nurse will report the reasons in the eCRF, i.e. patient participation withdrawal, refusal to complete the questionnaire without providing any reason, deterioration of health conditions precluding completion, lost to follow-up. During data analysis, as suggested by Fayers and Machin [Fayers 2000], reasons for failure to complete the questionnaire will be used as covariates in a logistic regression model, to investigate the association between compliance and indicated reasons.

9. MONITORING

Centralized trial monitoring will be carried out at the Research and Innovation Unit. During data collection, entered data will be systematically checked and a report will be prepared for each center, indicating expected questionnaires, questionnaires that were included, and those deemed not valid. All errors, incongruences, and omissions will be summarized in Data Query Forms, which will be sent to the investigators to elicit the necessary corrections. Investigators will be in charge of making the appropriate changes to the data of their center.

10. FEASIBILITY STUDY

Before the randomized trial begins, a pilot study will be performed at the cancer center of Cremona, on a consecutive sample of eligible patients who access the facility over two weeks (approximately 30 patients). This investigation aims to measure feasibility and acceptability of questionnaire

administration using a tablet. For this purpose, the following aspects will be recorded: the frequency of subjects declining participation in the survey and the corresponding reason (e.g. the use of a tablet), time taken by patients to complete the three instruments (HRQoL C30, HADS and NEQ), perceived difficulty and appreciation for the use of the tablet (expressed on a 5-point Likert scale [Abernethy 2009, Mark 2008]

11. Ethics and regulatory aspects

The study will be conducted in compliance with the principles of the revision of the Helsinki Statement and the legislation on scientific research. The protocol and patient informed consent will be submitted to the Ethics Committees (ECs)of the Hospital of Cremona and of all participating centers according to current Italian legislation. Before the trial is started at each center, authorization from the hospital's legal representative will be requested.

11.1 Informed consent

This study does not imply any type of experimental pharmacological treatment , or changes to diagnostic –therapeutic practice. Since the HQIS is implemented at each center regardless of patient informed consent signature, eligible patients will be asked to consent to the handling of their personal data. The informed consent form will be dated and signed by the patient and by the physician, authorized in accordance with regulations of the center's local EC. Information sheet and consent form for data handling will be used also for the feasibility study.
The informed consent form prepared by the coordinating center may be modified following specific requests of the local ECs. A copy of the signed informed consent shall be given to the patient and the original version retained by the center's investigator as part of the study documentation. It is the investigator's responsibility to obtain written informed consent to the use of health information. If a patient chooses not to continue with the study, it is the investigator's responsibility to ensure that no more health data of this person are collected. All gathered data will be used in the final analysis.

11.2 Confidentiality

All data collected, processed and stored for the purposes of the project will remain confidential at all times in compliance with GCP guidelines and current privacy regulations. Data will be gathered anonymously and sent to the study group in charge of data analysis and management, using a data communication system based on the HTTPs protocol (https://www.w3.org/2001/tag/doc/web-https) to ensure secure connections. Patients can only be identified by the clinical staff operating at the center where they were recruited. Each patient will receive personal credentials which he/she will use to complete the questionnaires. The research nurse will be in charge of sending the information to the central database. Backup will be performed daily on the central database.

12. ORGANIZATION AND RESPONSIBILITIES

12.1 Coordinating center

The trial will be coordinated by the Oncology Unit of the hospital of Cremona which assigns part of the activities to the Research and Innovation Unit of the University-Hospital of Parma, as follows: center recruitment and organization of training courses (Cremona), project management (Cremona), eCRF creation for tablet and training (Parma), data management and statistical data processing (Parma). The center of Cremona will also be in charge of obtaining the necessary authorizations to Ethics Committees and maintain contacts with clinical centers in the study.

12.2 Scientific Committee

The Scientific Committee (SC), also comprising independent members, is in charge of overall trial oversight and performs strategic guiding through periodic meetings during which it is informed by the coordination team on ongoing work, on any critical issues that have emerged and possible actions to undertake. The SC is responsible to decide whether a process must be stopped or modified.

12.3 Principal Investigators of participating centers

For each center, a study principal investigator is identified, in charge of:
- Be a liaison between the EC and center administration to ensure timely authorization
- Ensure efficient communication with the coordinating center
- Ensure adherence to the scientific protocol
- Ensure necessary recruitment
- Ensure adequate data collection and act as guarantor for data integrity and quality
- Ensure collaboration to solve any issues that may arise during the process of database quality control.
- Rapidly report to the Project manager any problems arisen during trial conduction at the center

12.4 Original document storing

Investigators shall keep at their center the documentation of each enrolled patient: documentation supporting eligibility and the original informed consent form signed by the patient. Investigators shall also prepare a folder (Investigator's File, IF) containing: all study documentation approved by the EC, including amendments and minutes, CRF, signed agreements, curriculum vitae of the investigator /s co-investigator/s, registry of enrolled subjects, list of subject identification codes. All documentation shall be kept for at least seven years after the end of the study, as required by current norms.

12.5 Publication of results

AIOM is the study promoter. The Unit of Cremona, the coordinating center, holds property of the database. Data will be published under the responsibility of the SC. Authorship will be determined by the SC by unanimous consensus and in accordance with usual rules for authorship attribution accepted internationally by the most authoritative journals, which essentially require fulfilment of the following criteria:

1) Substantial contributions to the conception or design of the work; or the acquisition, analysis, or interpretation of data for the work; 2) drafting the work or revising it critically for important intellectual content ; 3) final approval of the version to be published .

Author contribution to study design, enrolment, data analysis and manuscript drafting will therefore be considered for the definition of author order. Any request from the investigators to perform additional analyses for publications or conference presentations shall be submitted to the SC for evaluation and authorization. The SC shall receive a copy of every presentation, manuscript or abstract before dissemination.

12.6 Changes to the protocol

All amendments to the protocol shall be submitted to the ECs of participating centers. Amendments shall not be implemented before ethical approval is obtained. Changes merely concerning administrative or logistic aspects will be notified to the EC.

Should a violation to the protocol become necessary, the local study coordinator shall contact the principal investigator, possibly before the violation is implemented, or in any case as soon as possible, in order to discuss the matter and agree on the most appropriate course of action. Data reported in the CRF and in original documents will reflect all protocol violations and original documents will describe such violations and the circumstances that made them necessary.

12.7 Study duration

Overall expected duration is 30 months, with expected starting date in May 2016 and expected completion date in October 2018. Overall study conduction at the centers is 20 months (5 epochs of 4 months each), presumably from September 2016 (first data collection on patients) to December 2017 (last data collection on patients).

13. FUNDING AND COSTS

This study is funded by the Italian Association of Medical Oncology (AIOM), which will bear direct costs, i.e. for activities and additional resources required for study conduction at the centers. The study is co-financed by the Cremona no-profit Volunteer Association MEDeA (www.medeacremona.it , contributing €10.000/year. Co-financing covers all training costs, including travel, room and board expenses for instructors and participants, costs sustained by the coordination group and for the creation of the eCRF. These entities have no role in study design and conduction, data analysis and interpretation, or in the drafting of the manuscript and in the decision to submit it for publication.

14. REFERENCES

Abernethy AP, Herndon JE 2nd, Wheeler JL, Day JM, Hood L, Patwardhan M, Shaw H, Lyerly HK. Feasibility and acceptability to patients of a longitudinal system for evaluating cancer-related symptoms and quality of life: pilot study of an e/Tablet data-collection system in academic oncology. J Pain Symptom Manage. 2009Jun;37(6):1027-38.

Ament SM, Gillissen F, Moser A, Maessen JM, Dirksen CD, von Meyenfeldt MF, van der Weijden T. Identification of promising strategies to sustain improvements in hospital practice: a qualitative case study. BMC Health Serv Res. 2014 Dec 16;14:641.

American Society of Clinical Oncology. Outcomes of cancer treatment for technology assessment and cancer treatment guidelines. J Clin Oncol. 1996 Feb;14(2):671-9.

Annunziata MA, Muzzatti B, Altoè G. Defining hospital anxiety and depression scale (HADS) structure by confirmatory factor analysis: a contribution to validation for oncological settings. Ann Oncol. 2011 Oct;22(10):2330-3.

Apolone G, Filiberti A, Cifani S, Ruggiata R, Mosconi P. Evaluation of the EORTC QLQ-C30 questionnaire: a comparison with SF-36 Health Survey in a cohort of Italian long-survival cancer patients. Ann Oncol. 1998 May;9(5):549-57.

Aro K, Bäck L, Loimu V, Saarilahti K, Rogers S, Sintonen H, Roine R, Mäkitie
A. Trends in the 15D health-related quality of life over the first year following diagnosis of head and neck cancer. Eur Arch Otorhinolaryngol. 2015 Jul 28.

Arostegui I, Núñez-Antón V, Quintana JM. Statistical approaches to analyse patient-reported outcomes as response variables: an application to health-related quality of life. Stat Methods Med Res. 2012 Apr;21(2):189-214.

Au HJ, Ringash J, Brundage M, Palmer M, Richardson H, Meyer RM; NCIC CTG Quality of Life Committee. Added value of health-related quality of life measurement in cancer clinical trials: the experience of the NCIC CTG. Expert Rev Pharmacoecon Outcomes Res. 2010 Apr;10(2):119-28.

Basch E, Abernethy AP, Mullins CD, et al. Recommendations for Incorporating Patient-Reported Outcomes Into Clinical Comparative Effectiveness Research in Adult Oncology. J Clin Oncol 2012;30:4249-55

Berry DL, Blonquist TM, Patel RA, Halpenny B, McReynolds J. Exposure to a patient-centered, Web-based intervention for managing cancer symptom and quality of life issues: impact on symptom distress. J Med Internet Res. 2015 Jun
3;17(6):e136.

Brown RF, Bylund CL. Communication skills training: describing a new conceptual model. Acad Med 2008;83:37–44

Bultz BD, Carlson LE. Emotional distress: the sixth vital sign—future directions in cancer care. Psychooncology. 2006 Feb;15(2):93-5.

Bultz BD, Travado L, Jacobsen PB, et al. 2014 President's plenary international psycho-oncology society: moving toward cancer care for the whole patient. Psychooncology. 2015 Dec;24(12):1587-93.

Calvert M, Blazeby J, Altman DG, Revicki DA, Moher D, Brundage MD; CONSORT PRO Group. Reporting of patient-reported outcomes in randomized trials: the CONSORT PRO extension. JAMA. 2013 Feb 27;309(8):814-22.

Caminiti C, Diodati F, Filiberti S, Marcomini B, Annunziata MA, Ollari M, Passalacqua R. Cross-cultural adaptation and patients' judgments of a question prompt list for Italian-speaking cancer patients. BMC Health Serv Res. 2010 Jan 15;10:16.

Cella DF, Tulsky DS, Gray G, Sarafian B, Linn E, Bonomi A, Silberman M, Yellen SB, Winicour P, Brannon J, et al. The Functional Assessment of Cancer Therapy scale: development and validation of the general measure. J Clin Oncol. 1993 Mar;11(3):570-9.

Choi EP, Wong CK, Tsu JH, Chin WY, Kung K, Wong CK, Yiu MK. Health-related quality of life of Chinese patients with prostate cancer in comparison to general population and other cancer populations. Support Care Cancer. 2015 Oct 9.

Cocks K, King MT, Velikova G, Martyn St-James M, Fayers PM, Brown JM. Evidence-based guidelines for determination of sample size and interpretation of the European Organisation for the Research and Treatment of Cancer Quality of Life Questionnaire Core 30. J Clin Oncol. 2011 Jan 1;29(1):89-96.

Cocks K, King MT, Velikova G, de Castro G Jr, Martyn St-James M, Fayers PM, Brown JM. Evidence-based guidelines for interpreting change scores for the European Organisation for the Research and Treatment of Cancer Quality of Life Questionnaire Core 30. Eur J Cancer. 2012 Jul;48(11):1713-21.

Costantini M, Musso M, Viterbori P, Bonci F, Del Mastro L, Garrone O, Venturini M, Morasso G. Detecting psychological distress in cancer patients: validity of the Italian version of the Hospital Anxiety and Depression Scale.
Support Care Cancer. 1999 May;7(3):121-7.

Craig P, Dieppe P, Macintyre S, Michie S, Nazareth I, Petticrew M; Medical Research Council Guidance. Developing and evaluating complex interventions: the
new Medical Research Council guidance. BMJ. 2008 Sep 29;337:a1655.

European Partnership Action Against Cancer consensus group, Borras JM, Albreht T, Audisio R, Briers E, Casali P, Esperou H, Grube B, Hamoir M, Henning G, Kelly J, Knox S, Nabal M, Pierotti M, Lombardo C, van Harten W, Poston G, Prades J, Sant M, Travado L, Valentini V, van de Velde C, van den Bogaert S, van den Bulcke M, van Hoof E, van den Neucker I, Wilson R. Policy statement on multidisciplinary cancer care. Eur J Cancer. 2014 Feb;50(3):475-80.

Fallowfield L, Jenkins V, Farewell V, Solis-Trapala I. Enduring impact of communication skills training: results of a 12-month follow-up. Br J Cancer. 2003;89:1445–1449

Fayers and Machin . Quality of Life:Assessment, Analysis and Interpretation, J Wiley & Sons Ltd, Chicester, 2000, ISBN: 0-471-96861-7]

Galway K, Black A, Cantwell M, Cardwell CR, Mills M, Donnelly M. Psychosocial interventions to improve quality of life and emotional wellbeing for recently diagnosed cancer patients. Cochrane Database Syst Rev. 2012 Nov 14;11:CD007064.

Halyard MY, Ferrans CE. Quality-of-Life assessment for routine oncology clinical practice. J Support Oncol. 2008 May-Jun;6(5):221-9, 233.

Harrison JD, Young JM, Price MA, Butow PN, Solomon MJ. What are the unmet supportive care needs of people with cancer? A systematic review. Support Care Cancer. 2009 Aug;17(8):1117-28.

Hemming K, Girling A. The efficiency of stepped wedge vs. cluster randomized trials: stepped wedge studies do not always require a smaller sample size. J Clin Epidemiol. 2013 Dec;66(12):1427-8.

Hemming K, Girling A. A menu-driven facility for power and detectable-difference calculations in stepped-wedge cluster-randomized trials. Stat J. 2014;14 (2):363–380

Hemming K, Haines TP, Chilton PJ, Girling AJ, Lilford RJ. The stepped wedge cluster randomised trial: rationale, design, analysis, and reporting. BMJ. 2015 Feb 6;350:h391.

Hemming K, Lilford R, Girling AJ. Stepped-wedge cluster randomised controlled trials: a generic framework including parallel and multiple-level designs. Stat Med. 2015 Jan 30;34(2):181-96.

Hemming K, Taljaard M. Sample size calculations for stepped wedge and cluster randomised trials: a unified approach. J Clin Epidemiol. 2016 Jan;69:137-46.

Holland J, Watson M, Dunn J. The IPOS new International Standard of Quality Cancer Care: integrating the psychosocial domain into routine care. Psychooncology. 2011 Jul;20(7):677-80.

Howell D, Mayo S, Currie S, Jones G, Boyle M, Hack T, Green E, Hoffman L, Collacutt V, McLeod D, Simpson J; Canadian Association of Psychosocial Oncology (CAPO); Cancer Journey Action Group of the Canadian Partnership Against Cancer (CPAC). Psychosocial health care needs assessment of adult cancer patients: a consensus-based guideline. Support Care Cancer. 2012 Dec;20(12):3343-54.

Hussey MA, Hughes JP. Design and analysis of stepped wedge cluster randomized trials. Contemp Clin Trials. 2007 Feb;28(2):182-91.

Jacobsen PB, Wagner LI. A new quality standard: the integration of psychosocial care into routine cancer care. J Clin Oncol. 2012 Apr 10;30(11):1154-9.

Jacobsen PB, Lee M. Integrating Psychosocial Care Into Routine Cancer Care. Cancer Control. 2015 Oct;22(4):442-9. PubMed PMID: 26678971.

Johnson JR, Williams G, Pazdur R. End points and United States Food and Drug Administration approval of oncology drugs. J Clin Oncol. 2003 Apr 1;21(7):1404-11.

Kenzik KM, Ganz PA, Martin MY, Petersen L, Hays RD, Arora N, Pisu M. How much do cancer-related symptoms contribute to health-related quality of life in lung and colorectal cancer patients? A report from the Cancer Care Outcomes Research and Surveillance (CanCORS) Consortium. Cancer. 2015 Aug 15;121(16):2831-9.

Kesterke N, Egeter J, Erhardt JB, Jost B, Giesinger K. Patient-reported outcome assessment after total joint replacement: comparison of questionnaire completion times on paper and tablet computer. Arch Orthop Trauma Surg 2015;135:935–41.

Khan I, Bashir Z, Forster M. Interpreting small treatment differences from quality of life data in cancer trials: an alternative measure of treatment benefit and effect size for the EORTC-QLQ-C30. Health Qual Life Outcomes. 2015 Nov 14;13:180.

King MT, Bell ML, Costa D, Butow P, Oh B. The Quality of Life Questionnaire Core 30 (QLQ-C30) and Functional Assessment of Cancer-General (FACT-G) differ in responsiveness, relative efficiency, and therefore required sample size. J Clin Epidemiol. 2014 Jan;67(1):100-7.

Ledderer L, la Cour K, Mogensen O, Jakobsen E, Depont Christensen R, Kragstrup J, Hansen HP. Feasibility of a psychosocial rehabilitation intervention to enhance the involvement of relatives in cancer rehabilitation: pilot study for a randomized controlled trial. Patient. 2013;6(3):201-12.

Luckett T, King MT, Butow PN, Oguchi M, Rankin N, Price MA, Hackl NA, Heading G. Choosing between the EORTC QLQ-C30 and FACT-G for measuring health-related quality of life in cancer clinical research: issues, evidence and recommendations. Ann Oncol. 2011 Oct;22(10):2179-90.

Lundy JJ, Coons SJ, Aaronson NK. Testing the measurement equivalence of paper and interactive voice response system versions of the EORTC QLQ-C30. Qual Life Res. 2014 Feb;23(1):229-37.

Marcano Belisario JS, Jamsek J, Huckvale K, O'Donoghue J, Morrison CP, Car J. Comparison of self-administered survey questionnaire responses collected using mobile apps versus other methods. Cochrane Database Syst Rev. 2015 Jul 27;7:MR000042.

Mark TL, Fortner B, Johnson G. Evaluation of a tablet PC technology to screen and educate oncology patients. Support Care Cancer. 2008 Apr;16(4):371-8.

McLeod D, Curran J, Dumont S, White M, Charles G. The Interprofessional Psychosocial Oncology Distance Education (IPODE) project: perceived outcomes of an approach to healthcare professional education. J Interprof Care. 2014 May;28(3):254-9

Osoba D, Zee B, Pater J, Warr D, Kaizer L, Latreille J. Psychometric properties and responsiveness of the EORTC quality of Life Questionnaire (QLQ-C30) in patients with breast, ovarian and lung cancer. Qual Life Res. 1994 Oct;3(5):353-64.

Passalacqua R, Annunziata MA, Borreani C, Diodati F, Isa L, Saleri J, Verusio C, Caminiti C. Feasibility of a quality improvement strategy integrating psychosocial care into 28 medical cancer centers (HuCare project). Support Care Cancer. 2016 Jan;24(1):147-55.

Pollom EL, Wang E, Bui TT, Ognibene G, von Eyben R, Divi V, Sunwoo J, Kaplan M, Dimitri Colevas A, Le QT, Hara WY. A prospective study of electronic quality of life assessment using tablet devices during and after treatment of head and neck cancers. Oral Oncol. 2015 Dec;51(12):1132-7.

Roila F, Cortesi E. Quality of life as a primary end point in oncology. Ann Oncol. 2001;12 Suppl 3:S3-6.

Stanton AL. Psychosocial concerns and interventions for cancer survivors. J Clin Oncol. 2006 Nov 10;24(32):5132-7.

Tamburini M, Gangeri L, Brunelli C, Beltrami E, Boeri P, Borreani C, Fusco Karmann C, Greco M, Miccinesi G, Murru L, Trimigno P. Assessment of hospitalized cancer patients' needs by the Needs Evaluation Questionnaire. Ann Oncol. 2000 Jan;11(1):31-7.

Uwer L, Rotonda C, Guillemin F, Miny J, Kaminsky MC, Mercier M, Tournier-Rangeard L, Leonard I, Montcuquet P, Rauch P, Conroy T. Responsiveness of EORTC QLQ-C30, QLQ-CR38 and FACT-C quality of life questionnaires in patients with colorectal cancer. Health Qual Life Outcomes. 2011 Aug 22;9:70.

Vodermaier A, Millman RD. Accuracy of the Hospital Anxiety and Depression Scale as a screening tool in cancer patients: a systematic review and meta-analysis. Support Care Cancer. 2011 Dec;19(12):1899-908.

Zhu L, Ranchor AV, van der Lee M, Garssen B, Sanderman R, Schroevers MJ. Subtypes of depression in cancer patients: an empirically driven approach. Support Care Cancer. 2016 Mar;24(3):1387-96.

ATTACHMENT I - FACILITIES QUESTIONNAIRE

Il presente progetto, promosso dall'Associazione Italiana Oncologia Medica, segue l'attuazione dello studio HuCare, finanziato nel 2009 dal Ministero della Salute e dalla Regione Lombardia e i cui risultati sono stati pubblicati di recente sulla rivista Supportive Care in Cancer. Lo studio ha dimostrato che è possibile trasferire interventi psicosociali "evidence-based" nella pratica clinica oncologica, attuando un'adeguata strategia di implementazione e garantendo il coinvolgimento di tutta l'equipe clinica (medici, infermieri, psicologi).
Il progetto HuCare2 ha l'obiettivo di valutare se l'applicazione di questa strategia, quindi di un set di interventi psicosociali, consente di migliorare la qualità di vita dei pazienti che iniziano un trattamento attivo. Il questionario è volto a verificare la presenza nel centro delle caratteristiche necessarie alla realizzazione degli interventi previsti dal progetto e a determinarne l'eleggibilità.
Vi preghiamo pertanto di rispondere alle seguenti domande, indicando, in caso di risposta negativa, le barriere all'introduzione dell'intervento, selezionando tra quelle indicate, tratte dalla letteratura, o specificando i motivi nel campo apposito se non presente nell'elenco.

____

Data presentazione del progetto a tutto il personale sanitario dell'U.O. __/___/____

1. Il Progetto prevede che tutto il personale medico svolga un corso residenziale di 3 gg per migliorare le proprie capacità comunicative. Tutte le edizioni dei corsi avranno luogo a Milano, presso la scuola di perfezionamento in comunicazione oncologica (Scuola AIOM-HuCare) situata nel Palazzo Stelline, nell'arco di due mesi (periodo assegnato al suo centro in base alla randomizzazione, vedi sinossi studio). I costi della formazione saranno coperti con i fondi del progetto, comprese le spese di trasferta, vitto e alloggio.
Lei conferma la possibilità che almeno il 75% dei medici oncologi (dipendenti e contrattisti a tempo determinato) del Suo reparto possano partecipare a tale corso?

[ ] SI [ ] NO [ ] SOLO IN PARTE/PARZIALMENTE A CAUSA DI:
[ ] carico di lavoro eccessivo, organizzazione del lavoro e indisponibilità di tempo
[ ] disaccordo tra i membri dell'equipe
[ ] mancanza di riconoscimento da parte della Direzione aziendale
[ ] mancanza di consapevolezza dell'importanza
[ ] mancanza di fiducia nell'efficacia dell'iniziativa

[ ] altro ____

____

____

2. Il Progetto prevede che tutto il personale infermieristico svolga un corso di formazione di 2 gg, per migliorare le capacità comunicative/relazionali e per gestire il PIS. Tutte le edizioni dei corsi avranno luogo a Milano, presso la scuola di perfezionamento in comunicazione oncologica (Scuola AIOM-HuCare) situata nel Palazzo Stelline, nel periodo assegnato al suo centro in base alla randomizzazione (vedi descrizione studio). I costi della formazione saranno a carico dei fondi del progetto, comprese le spese di trasferta, vitto e alloggio.
Lei conferma la possibilità che almeno il 75% degli infermieri del Suo reparto possano partecipare a tale corso?

[ ] SI [ ] NO [ ] SOLO IN PARTE/PARZIALMENTE A CAUSA DI:
[ ] carico di lavoro eccessivo, organizzazione del lavoro e indisponibilità di tempo
[ ] disaccordo tra i membri dell’equipe
[ ] mancanza di riconoscimento da parte della Direzione aziendale
[ ] mancanza di consapevolezza dell’importanza
[ ] mancanza di fiducia nell’efficacia dell’iniziativa

[ ] altro ____

____

____

3. Il Progetto prevede l'introduzione di un percorso informativo e di accoglienza per tutti i nuovi pazienti prima dell'inizio della terapia. Tale percorso presuppone il coinvolgimento attivo del personale infermieristico e implica alcuni cambiamenti organizzativi.
Lei conferma la disponibilità del personale del Suo reparto ad introdurre questo nuovo percorso, illustrato nella breve descrizione delle attività del progetto allegata?

[ ] SI [ ] NO [ ] SOLO IN PARTE/PARZIALMENTE A CAUSA DI:
[ ] carico di lavoro eccessivo, organizzazione del lavoro e indisponibilità di tempo
[ ] disaccordo tra i membri dell’equipe
[ ] mancanza di riconoscimento da parte della Direzione aziendale
[ ] mancanza di consapevolezza dell’importanza
[ ] mancanza di fiducia nell’efficacia dell’iniziativa

[ ] altro ____

____

____

4. Il Progetto prevede la disponibilità in ogni reparto di un luogo, anche di piccole dimensioni, da adibire all'allestimento del PIS (Punto di Informazione e Supporto) con le seguenti caratteristiche:

- Attiguo al DH e/o agli ambulatori del reparto di oncologia
- Possibilità di accesso a Internet
- Disponibilità di scaffale o armadio da allestire per biblioteca
- Garanzia di privacy per lo svolgimento del colloquio infermiere-paziente.

Lei conferma la disponibilità di allestire tale struttura nella sua U.O.?

[ ] SI [ ] NO [ ] SOLO IN PARTE/PARZIALMENTE A CAUSA DI:

[ ] mancanza di risorse economiche per acquisire gli arredi
[ ] mancanza di autorizzazione della Direzione
[ ] mancanza di spazi

[ ] altro ____

____

____

5. Il Progetto prevede che un infermiere di riferimento del paziente fornisca il supporto informativo in base alle necessità rilevate con un questionario.
Lei conferma la possibilità che gli infermieri nel Suo reparto svolgano questa funzione?

[ ] SI [ ] NO [ ] SOLO IN PARTE/PARZIALMENTE A CAUSA DI:

[ ] carico di lavoro eccessivo, organizzazione del lavoro e indisponibilità di tempo
[ ] mancanza di riconoscimento da parte della Direzione aziendale
[ ] mancanza di consapevolezza dell'importanza
[ ] mancanza di fiducia nell'efficacia dell'iniziativa
[ ] mancanza di riconoscimento del ruolo da parte dell'equipe
[ ] mancanza di riconoscimento economico

[ ] altro ____

____

____

6. Il Progetto prevede la disponibilità in ogni reparto di uno psicologo per almeno 2 giorni la settimana. Lei conferma la disponibilità di tale figura nella sua U.O.?

[ ] SI [ ] NO [ ] SOLO IN PARTE/PARZIALMENTE A CAUSA DI:

[ ] indisponibilità di risorse economiche
[ ] mancanza di supporto della Direzione

[ ] altro ____

____

____

7. Il Progetto prevede l'affidamento dei pazienti che ne hanno bisogno a un assistente sociale Lei conferma la disponibilità di tale figura nel suo ospedale?

[ ] SI [ ] NO

Note:

____

____

Descrizione dell'Unità Operativa

Direttore reparto……… ………………………………………………………………………..…………………
Istituzione ……………………………………………………………...………………………...……………………
Reparto………………………………………………………………………………..………………………………
Indirizzo………………………………………………………………………………………………..………
CAP…..……… Città………………………………………………………………… Provincia ……………………
Telefono …………………………
E-mail …………………………………………………..……………………………………………………………

Numero Medici Oncologi (dipendenti e contrattisti a tempo determinato), incluso il Direttore ……

Numero Infermieri professionali, incluso il Coordinatore ……

Il Suo centro ha già un percorso prestabilito e strutturato per informare i pazienti?

SI [ ] NO [ ] IN PARTE [ ]

Nel Suo centro vengono routinariamente rilevati i bisogni psicologici dei pazienti?

SI [ ] NO [ ] IN PARTE [ ]

Se sì, con quale strumento? _______________________________________

Nel Suo centro vengono routinariamente rilevati i bisogni sociali dei pazienti (assistenza domiciliare, accompagnamento dei pazienti, supporto finanziario, ecc)?

SI [ ] NO [ ] IN PARTE [ ]

Se sì, con quale strumento?____

Figure di Riferimento del Centro

Medico Referente del progetto
Nome e cognome ____
Email ____
Tel ____

Coordinatore infermieristico
Nome e cognome ____
Email ____
Tel ____

Infermiere referente (responsabile della somministrazione del questionario sulla qualità di vita)
Nome e cognome ____
Email ____
Tel ____

Psicologo clinico di riferimento
Nome e cognome ____
Email ____
Tel ____
Tipo di contratto ____
N. ore/settimana di presenza in reparto _____________

Note ____

____

L'adesione comporta la partecipazione globale a tutte le iniziative del Progetto e non può essere limitata soltanto a una o alcune delle sue fasi.

Confermo la mia intenzione, del Coordinatore Infermieristico e quella di tutto lo staff a partecipare al Progetto HUCARE2.

Firma del Direttore/Responsabile ....………...

Data ....

ATTACHMENT II – AGENDAS OF TRAINING COURSES

Training Course for Physicians "Communication with the cancer patient"

Day 1

- Introduction of participants
- Introduction
- Psychooncology
- Break
- Communication in oncology
- Discussion
- Lunch
- Role-playing with actors performing patients, on cases presented by the staff, videotaped and followed by debate
- Discussion

For the following day, participants will identify a critical case from their clinical practice

Day 2

- Communication through the different stages of illness
- Discussion
- Break
- Videotape viewing followed by debate
- Lunch
- Role-playing of clinical cases presented by participants, followed by debate
- Discussion

**Day 3**

- The family
- Discussion
- Role-playing of clinical cases presented by participants followed by debate
- Break
- Group work on specific topics
- Lunch
- Group reporting, plenary discussion
- Final test and course evaluation questionnaire

Training Course for nurses: “Relationship with the cancer patient”

**Day 1**

- Introduction of participants
- Analysis of a critical clinical case
- Lecture: “Cancer’s emotional impact and the needs of patients and families”
- Lecture: “Communication skills: active listening; empathy; use of questions; re-assurance
- Break
- Exercises: “active listening”
- Lecture: “non-verbal communication”
- Exercises “Non-verbal communication”
- Lunch
- Role-playing of critical cases followed by debate

**Day 2**

- The importance of information in oncology
- The role of nurses in information: competences and autonomy
- Conflicts nurses face in the patient information process
- Break
- Information: products and tools
- Main Italian and foreign web resources
- Lunch
- Data collection sheet and database
- Role-playing of patient information requests, followed by debate
- Final discussion

ATTACHMENT III - QUESTION PROMPT LIST

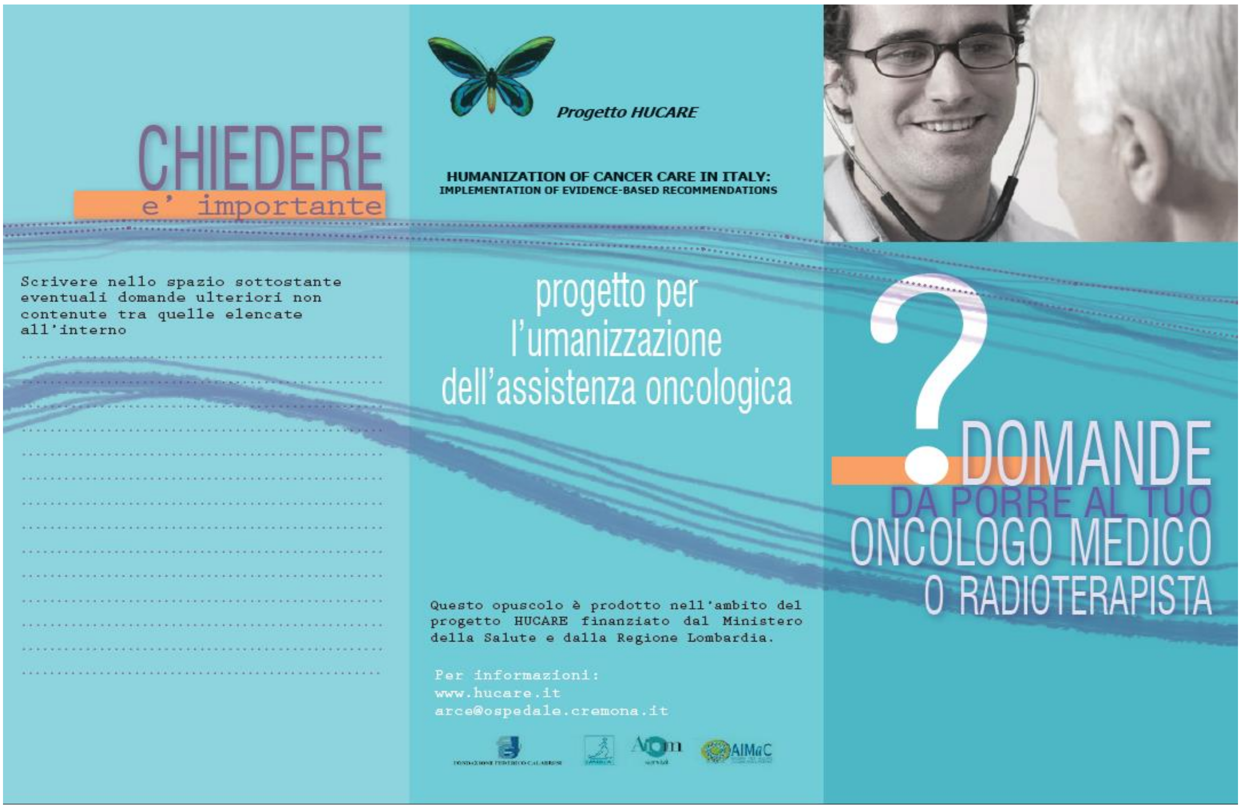

Quando oggi incontrerà il suo medico probabilmente avrà domande e dubbi, che è facile dimenticare nella tensione del momento per poi ricordarsene solo in seguito. Per aiutarla ad ottenere tutte le informazioni di cui ha bisogno circa la sua malattia e le possibili terapie, abbiamo compilato un elenco di domande. Quelle che seguono sono le domande che più frequentemente i pazienti rivolgono ai medici, lei può scegliere di utilizzare alcune di queste domande o nessuna. Probabilmente, molte di esse non la riguardano, o non la riguardano in questo momento. Lei e i suoi familiari potrete decidere di utilizzare questo elenco in qualsiasi momento.
Le suggeriamo di barrare le domande che intende porre, e di annotarsi quelle che le vengono in mente, ma non sono contenute nell'elenco. Sappia che è sua facoltà chiedere e che troverà il medico disposto a rispondere e ad esaudire i suoi bisogni informativi.

COME E QUANDO CHIEDERE
- ☐ Oggi ha un po' di tempo per rispondere alle mie domande?
- ☐ Posso chiederle di spiegarmi il significato di termini che non conosco bene?

DIAGNOSI
- ☐ Che tipo di tumore ho?
- ☐ Dove si trova il tumore adesso? Si è esteso ad altre parti del corpo?
- ☐ Quanto è comune la mia malattia?

ESAMI
- ☐ Devo fare altri esami? Quali altre informazioni ci daranno? Confermeranno la mia diagnosi?
- ☐ Cosa mi succederà durante questo/i esame/i?

PROGNOSI
- ☐ Quanto è grave questo tumore e cosa significherà per me?
- ☐ Quali saranno i sintomi?
- ☐ Qual è l'obiettivo della terapia? Guarirmi dal tumore o tenerlo sotto controllo e alleviare i sintomi?
- ☐ Senza ulteriori terapie, qual è la probabilità che il tumore si estenda ad altre parti del corpo?
- ☐ Se decido di sottopormi ad ulteriori terapie, qual è la probabilità che il tumore si estenda ad altre parti del corpo?
- ☐ Qual è l'aspettativa di vita per chi ha il mio stesso tumore?
- ☐ Esistono servizi-gruppi di sostegno che potrebbero aiutare me e i miei familiari ad affrontare questa malattia?
- ☐ La terapia migliorerà le mie probabilità di sopravvivenza?
- ☐ Qual è la probabilità che la terapia migliori i miei sintomi? Vale la pena affrontarla?
- ☐ E' possibile che la terapia, o la malattia, riducano il mio desiderio sessuale?

ASSICURARSI LE CURE MIGLIORI
- ☐ Lei è specializzato nel trattamento di un tumore come il mio?
- ☐ Quanto è utilizzata e consolidata la terapia che mi consiglia?
- ☐ Ci sono linee guida per il trattamento della mia malattia, cioè documenti che contengono raccomandazioni su diagnosi e terapie di questo tipo di tumore, su cui concorda la maggior parte di esperti?
- ☐ Conosce qualche altro specialista che tratta il mio tipo di tumore a cui potrei rivolgermi per una seconda opinione?

L'EQUIPE MULTIDISCIPLINARE
- ☐ Lei lavora in una equipe multidisciplinare, cioè collabora con colleghi specializzati in diversi aspetti della mia malattia?
- ☐ Può spiegarmi i vantaggi di un lavoro di equipe, cioè della collaborazione con colleghi specializzati nella mia malattia?
- ☐ Come si svolgerà la comunicazione tra di voi e tra voi e me?
- ☐ Chi mi seguirà nel percorso di cura?
- ☐ Come mi devo comportare se mi vengono date informazioni contrastanti?

INFORMAZIONI E OPZIONI TERAPEUTICHE
Opzioni
- ☐ E' proprio necessario cominciare la terapia ora?
- ☐ Se sì, posso scegliere tra diversi trattamenti?
- ☐ Quali sono i pro e i contro di ciascuna opzione terapeutica?
- ☐ Cosa posso aspettarmi nel caso decidessi di non sottopormi ad alcun trattamento?
- ☐ Quanto tempo ho per pensarci? Devo decidere oggi stesso?
- ☐ Secondo lei qual è la migliore terapia per il mio caso?

Terapia
- ☐ Come si svolgerà esattamente la terapia e che effetti avrà su di me? Presumibilmente quando si verificheranno questi effetti?
- ☐ Qual è il programma terapeutico che dovrò seguire, es. quanti cicli di terapia, con che frequenza, e per quanto tempo?
- ☐ Dove farò la terapia?
- ☐ Ci sono vantaggi/svantaggi tra strutture private e pubbliche?

STUDI CLINICI
- ☐ Ci sono studi in corso che potrebbero applicarsi al mio caso?
- ☐ Sarei curato diversamente se prendessi parte a uno studio clinico?

PREPARAZIONE ALLA TERAPIA
- ☐ C'è qualcosa che potrei fare prima o dopo la terapia per renderla più efficace, (alimentazione, lavoro, esercizio fisico, ecc.)?
- ☐ Quali sono le cose che si possono o non si possono fare durante la terapia?
- ☐ Che problemi potrebbero insorgere e chi dovrò contattare se si verificheranno?
- ☐ La terapia comporta effetti collaterali a lungo termine?
- ☐ Dovrò sottopormi ad altri trattamenti dopo questo? Se sì, quali?
- ☐ Che controlli dovrò fare dopo la fine della terapia?

COSTI
- ☐ Che spese dovrò sostenere durante tutto il trattamento (es. farmaci, chemioterapia, ecc.?)
- ☐ Avrò diritto a qualche indennità se non potrò lavorare?

MATERIALE INFORMATIVO
- ☐ Che informazioni esistono relativamente al mio tumore e al suo trattamento (libri, video, siti internet, ecc.)?
- ☐ Esistono terapie alternative che lei ritiene potrebbero essere utili o dannose in casi come il mio?
- ☐ E' possibile parlare con qualcuno che si è sottoposto a questa terapia?
- ☐ Esistono servizi-gruppi di sostegno che potrebbero aiutare me e i miei familiari ad affrontare questa malattia?

ATTACHMENT IV – DATA COLLECTION FORM

Centro	Paziente n°
|__|__|__|	|__|__|__|

SCHEDA RACCOLTA DATI

Trial clinico controllato e randomizzato, con disegno a cluster stepped wedge, per valutare una strategia volta ad ottimizzare gli outcomes psicosociali in pazienti affetti da cancro

	Centro	Paziente n°
VISITA DI SCREENING – Eleggibilità del paziente	|__|__|__|	|__|__|__|

Criteri di inclusione

		Si [1]	No [2]
1.	Età ≥ 18 e ≤ 75 anni	○	○
2.	Diagnosi (istologica o citologica) di tumore solido da non più di un mese	○	○
3.	Deve iniziare un primo trattamento medico: chemioterapia EV, farmaci orali, farmaci a target molecolari	○	○
4.	Sopravvivenza attesa > 3 mesi	○	○
5.	Buona comprensione della lingua italiana	○	○

Criteri di esclusione

		Si [1]	No[2]
1.	Pregressa chemioterapia o altro trattamento medico per tumore	○	○
2.	Arruolato nel medesimo trial in un periodo precedente	○	○
3.	Partecipazione in atto ad altri trial che prevedono la misura di PRO	○	○
4.	In trattamento da uno psicologo o psichiatra o con antidepressivi	○	○
5.	Ricoverato in degenza ordinaria	○	○
6.	Presenza di condizioni patologiche mentali o psichiatriche, dovute al tumore o a patologie concomitanti, che interferiscono con lo stato di coscienza o con la capacità di giudizio	○	○
7.	Impossibilità a completare il questionario o a garantire il follow-up a tre mesi	○	○

CONSENSO INFORMATO

Se il soggetto è eleggibile, si prega di informarlo esaustivamente sulle finalità dello studio, di far firmare l'apposito modulo del consenso informato scritto e di riportare qui di fianco la data della firma:

|__|__|__|__|__|__|__|__|
g g m m a a a a

VALUTAZIONE BASALE - Caratteristiche demografiche paziente	Centro	Paziente n°
	|__|__|__|	|__|__|__|

1. Data di nascita \|__\|__\|__\|__\|__\|__\|__\|__\|
g g m m a a a a

2. Sesso ○ **Uomo [1]** ○ **Donna [2]**

3. Stato civile ○ **Coniugato/a o convive stabilmente [1]** ○ **Altro [2]**

4. Livello d'istruzione massimo
○ **Primario (nessuno/ elementare/ scuola media) [1]**
○ **Secondario (diploma scuola media superiore) [2]**
○ **Universitario (laurea o post laurea) [3]**

VALUTAZIONE BASALE – Caratteristiche cliniche paziente	Centro	Paziente n°
	|_|_|_|	|_|_|_|

1. Data rilevazione |__|__|__|__|__|__|__|__|
g g m m a a a a

Data della prima diagnosi del tumore primitivo 2. |__|__| m m 3. |__|__|__|__| a a a a

4. Tumore metastatico: ○ **Si** ○ **No**

5. Sede del tumore primitivo

Polmone	○ [1]	Stomaco ed esofago	○ [7]
Colon e retto	○ [2]	Capo e collo	○ [8]
Mammella	○ [3]	Ginecologici	○ [9]
Prostata	○ [4]	Fegato e vie biliari	○ [10]
Pancreas	○ [5]	Altra sede / Sede non nota	○ [11]
Apparato urinario	○ [6]		

Se TUMORE METASTATICO, indicare le sedi:

	Si [1]	No [2]
5.1 Epatica	○	○
5.2 Polmonare	○	○
5.3 Ossea	○	○
5.4 Cerebrale	○	○
5.5 Addominale	○	○
5.6 Linfonodale	○	○
5.7 Altra sede (specificare)	○	○
5.8 ________________________		

6. Performance Status di ECOG: 0 ○ 1 ○ 2 ○ 3 ○ 4 ○ 5 ○

7. Tipo di trattamento: ○ **Chemioterapia EV [1]**
○ **Farmaci orali [2]**
○ **Farmaci a target molecolare [3]**

VALUTAZIONE BASALE – Qualità della vita	Centro	Paziente n°
	|_|_|_|	|_|_|_|

ITALIAN

EORTC QLQ-C30 (versione 3)

Con questo questionario vorremmo sapere alcune cose su di Lei e sulla Sua salute. La preghiamo di rispondere a tutte le domande ponendo un cerchio attorno al numero che meglio corrisponde alla Sua risposta. Non esiste una risposta "giusta" o "sbagliata". Le Sue informazioni verranno tenute strettamente riservate.

Per favore scriva solo le iniziali del Suo nome e cognome: |_|_|_|_|
Data di nascita (g, m, a): |_|_|_|_|_|_|_|_|
La data di oggi (g, m, a): 31 |_|_|_|_|_|_|_|_|

	No	Un po'	Parec-chio	Moltis-simo
1. Ha difficoltà nel fare lavori faticosi, come portare una borsa della spesa pesante o una valigia?	1	2	3	4
2. Ha difficoltà nel fare una lunga passeggiata?	1	2	3	4
3. Ha difficoltà nel fare una breve passeggiata fuori casa?	1	2	3	4
4. Ha bisogno di stare a letto o su una sedia durante il giorno?	1	2	3	4
5. Ha bisogno di aiuto per mangiare, vestirsi, lavarsi o andare in bagno?	1	2	3	4

**Durante gli ultimi sette giorni:**	No	Un po'	Parec-chio	Moltis-simo
6. Ha avuto limitazioni nel fare il Suo lavoro o i lavori di casa?	1	2	3	4
7. Ha avuto limitazioni nel praticare i Suoi passatempi-hobby o altre attività di divertimento o svago?	1	2	3	4
8. Le è mancato il fiato?	1	2	3	4
9. Ha avuto dolore?	1	2	3	4
10. Ha avuto bisogno di riposo?	1	2	3	4
11. Ha avuto difficoltà a dormire?	1	2	3	4
12. Ha sentito debolezza?	1	2	3	4
13. Le è mancato l'appetito?	1	2	3	4
14. Ha avuto un senso di nausea?	1	2	3	4
15. Ha vomitato?	1	2	3	4
16. Ha avuto problemi di stitichezza?	1	2	3	4

Continuare alla pagina successiva

VALUTAZIONE BASALE – Qualità della vita	Centro	Paziente n°
	|_|_|_|	|_|_|_|

ITALIAN

Durante gli ultimi sette giorni:	No	Un po'	Parec-chio	Moltis-simo
17. Ha avuto problemi di diarrea?	1	2	3	4
18. Ha sentito stanchezza?	1	2	3	4
19. Il dolore ha interferito con le Sue attività quotidiane?	1	2	3	4
20. Ha avuto difficoltà a concentrarsi su cose come leggere un giornale o guardare la televisione?	1	2	3	4
21. Si è sentito(a) teso(a)?	1	2	3	4
22. Ha avuto preoccupazioni?	1	2	3	4
23. Ha avuto manifestazioni di irritabilità?	1	2	3	4
24. Ha avvertito uno stato di depressione?	1	2	3	4
25. Ha avuto difficoltà a ricordare le cose?	1	2	3	4
26. Le Sue condizioni fisiche o il Suo trattamento medico hanno interferito con la Sua vita familiare?	1	2	3	4
27. Le Sue condizioni fisiche o il Suo trattamento medico hanno interferito con le Sue attività sociali?	1	2	3	4
28. Le Sue condizioni fisiche o il Suo trattamento medico Le hanno causato difficoltà finanziarie?	1	2	3	4

Per le seguenti domande ponga un cerchio intorno al numero da 1 a 7 che meglio corrisponde alla Sua risposta

29. Come valuterebbe in generale la Sua salute durante gli ultimi sette giorni?

1	2	3	4	5	6	7
Pessima						Ottima

30. Come valuterebbe in generale la Sua qualità di vita durante gli ultimi sette giorni?

1	2	3	4	5	6	7
Pessima						Ottima

© QLQ-C30 Copyright 1995 EORTC Quality of Life Group. Tutti i diritti sono riservati. Versione 3.0

VALUTAZIONE BASALE – Depressione e ansia	Centro	Paziente n°
	|_|_|_|	|_|_|_|

Questionario sulla depressione e sull'ansia durante la degenza ospedaliera (HADS)

GL assessment
the measure of potential

Nome: ______________________________ Data: ______________

I medici sono consapevoli che le emozioni rivestono un ruolo importante nella maggior parte delle malattie. Se il suo medico conosce le sue sensazioni, gli sarà più facile aiutarla.

Questo questionario ha lo scopo di aiutare il suo medico a capire come lei si sente. Legga ciascuna domanda riportata qui sotto e **sottolinei la risposta** che più si avvicina a come lei si è sentito/a negli ultimi 7 giorni. Ignori i numeri stampati ai margini del questionario.

Risponda alle domande senza pensarci troppo, la sua reazione immediata a ciascuna domanda sarà probabilmente più precisa di una risposta su cui ha riflettuto a lungo.

Mi sono sentito/a teso/a o molto nervoso/a
- Quasi sempre
- Spesso
- A volte
- Mai

Ho continuato a provare piacere per le stesse cose che mi piacevano prima
- Proprio come prima
- Non proprio come prima
- Solo in parte
- Quasi per niente

Ho provato una sensazione di paura come se stesse per accadere qualcosa di terribile
- Sicuramente e tanto
- Sì, ma non tanto
- Un po', ma non da preoccuparmene
- Mai

Sono riuscito/a a ridere e a vedere il lato divertente delle cose
- Proprio come ho sempre fatto
- Non proprio come prima
- Sicuramente non come prima
- Per niente

Mi sono venuti in mente pensieri preoccupanti
- Quasi sempre
- Spesso
- A volte
- Quasi mai

Mi sono sentito/a di buon umore
- Mai
- Raramente
- A volte
- Quasi sempre

Sono riuscito/a a stare seduto fermo/a e a sentirmi rilassato/a
- Sempre
- Spesso
- Raramente
- Mai

Mi sono sentito/a come rallentato/a
- Quasi sempre
- Molto spesso
- A volte
- Mai

Ho provato una sensazione di paura, come un senso di tensione allo stomaco
- Mai
- A volte
- Spesso
- Molto spesso

Ho perso interesse per il mio aspetto fisico
- Completamente
- Spesso non me ne prendo cura quanto dovrei
- A volte non me ne prendo cura abbastanza
- Me ne prendo cura come al solito

Mi sono sentito/a irrequieto/a e incapace di stare fermo/a
- Moltissimo
- Molto
- Non molto
- Mai

Ho pensato al futuro con ottimismo
- Come sempre
- Un po' meno di prima
- Molto meno di prima
- Quasi per niente

Ho avuto improvvise sensazioni di panico
- Molto spesso
- Spesso
- Raramente
- Mai

Sono riuscito/a a godermi un buon libro o un buon programma alla radio o alla televisione
- Spesso
- A volte
- Raramente
- Molto raramente

Ora controlli di aver risposto a tutte le domande

TOTALE

	Centro	Paziente n°
VALUTAZIONE BASALE – Bisogni dei pazienti	|__|__|__|	|__|__|__|

Questionario per la Valutazione dei Bisogni del Paziente

(NEQ)

Gent.le Signora/Gent.le Signore

Il seguente questionario riporta un elenco di bisogni, legati alla condizione di salute, che alcune persone hanno detto di avere.

Le chiediamo di rispondere **SI** per i bisogni che Lei ritiene di avere **in questo momento** e **NO** per i bisogni che pensa di non avere.

		SI [1]	NO [2]
**A**	"Ho bisogno di avere maggiori informazioni sulla mia diagnosi"	◎	◎
**B**	"Ho bisogno di avere maggiori informazioni sulle mie condizioni future"	◎	◎
**C**	"Ho bisogno di avere maggiori informazioni sugli esami che mi stanno facendo"	◎	◎
**D**	"Ho bisogno di avere maggiori spiegazioni sui trattamenti"	◎	◎
**E**	"Ho bisogno di essere più coinvolto/a nelle scelte terapeutiche"	◎	◎
**F**	"Ho bisogno che i medici e gli infermieri mi diano informazioni più comprensibili"	◎	◎
**G**	"Ho bisogno che i medici siano più sinceri con me"	◎	◎
**H**	"Ho bisogno di avere un dialogo maggiore con i medici"	◎	◎
**I**	"Ho bisogno che alcuni dei miei disturbi (dolore, nausea, insonnia,ecc.) siano maggiormente controllati"	◎	◎
**L**	"Ho bisogno di maggiore aiuto per mangiare, vestirmi ed andare in bagno"	◎	◎
**M**	"Ho bisogno di maggiore rispetto della mia intimità"	◎	◎
**N**	"Ho bisogno di maggiore attenzione da parte del personale infermieristico"	◎	◎
**O**	"Ho bisogno di essere più rassicurato dai medici"	◎	◎
**P**	"Ho bisogno che i servizi offerti dall'ospedale (bagni, pasti, pulizia) siano migliori"	◎	◎
**Q**	"Ho bisogno di avere maggiori informazioni economico-assicurative legate alla mia malattia (ticket, invalidità, ecc.)"	◎	◎
**R**	"Ho bisogno di un aiuto economico"	◎	◎
**S**	"Ho bisogno di parlare con uno psicologo"	◎	◎
**T**	"Ho bisogno di parlare con un assistente spirituale"	◎	◎
**U**	"Ho bisogno di parlare con persone che hanno avuto la mia stessa esperienza"	◎	◎
**V**	"Ho bisogno di essere maggiormente rassicurato dai miei famigliari"	◎	◎

**VALUTAZIONE BASALE – Bisogni dei pazienti**	Centro: └─┴─┴─┘ · Paziente n°: └─┴─┴─┘

**X**	"Ho bisogno di sentirmi maggiormente utile in famiglia"	○	○
**Y**	"Ho bisogno di sentirmi meno abbandonato a me stesso"	○	○
**Z**	"Ho bisogno di essere meno commiserato dagli altri"	○	○

Altro:

____

____

In questo momento il mio bisogno principale è:

____

____

VALUTAZIONE BASALE – Motivi della non compilazione	Centro	Paziente n°
	|__|__|__|	|__|__|__|

REPORTING FORM

Compilazione a cura dell'infermiere di ricerca

1. Se il paziente non ha compilato tutti e 3 i questionari o almeno uno dei questionari presenta meno del 50% degli item, indicare la motivazione:

- ◎ **Si rifiuta di completare il questionario [1]**
- ◎ **Le condizioni di salute non consentono di proseguire[2]**
- ◎ **Altro [3], specificare [3.1] __________________________________**

VALUTAZIONE FOLLOW-UP – a 3 mesi dall’arruolamento	Centro	Paziente n°
	|__|__|__|	|__|__|__|

VALUTAZIONE DELLA QUALITA DELLA VITA CON EORTC QLQ-C30

VALUTAZIONE DELLA DEPRESSIONE E DELL’ANSIA CON HADS

VALUTAZIONE DEI BISOGNI PSICOSOCIALI CON NEQ

VALUTAZIONE FOLLOW-UP – a 3 mesi dall'arruolamento	Centro	Paziente n°
	|__|__|__|	|__|__|__|

REPORTING FORM

Compilazione a cura dell'infermiere di ricerca

1. Performance Status di ECOG: ○ 0 ○ 1 ○ 2 ○ 3 ○ 4 ○ 5

2. Il paziente è in progressione?

○ Si [1] ○ No [2] ○ Non applicabile [3]

1. Se il paziente non ha compilato tutti e 3 i questionari o almeno uno dei questionari presenta meno del 50% degli item, indicare la motivazione:

○ Il paziente ha ritirato l'adesione allo studio [1]

○ Si rifiuta di compilare il questionario [2]

○ Le condizioni di salute non consentono la compilazione [3]

○ Perso al follow-up [4]

○ Deceduto [5]

○ Altro [6], specificare [6.1]______________________________

STUDIO DI FATTIBILITÀ	Centro	Paziente n°
	|__|__|__|	|__|__|__|

SCHEDA RACCOLTA DATI

Indagine pilota per misurare il gradimento dei pazienti e la fattibilità dell'utilizzo di un dispositivo informatico (tablet) per la misura della Qualità di Vita e dei bisogni psicosociali

STUDIO DI FATTIBILITÀ	Centro	Paziente n°
	|__|__|__|	|__|__|__|

Durante la fase di screening, nel caso in cui il paziente rifiuta la partecipazione allo studio, chiedere:

Quale è la motivazione per cui non intende partecipare allo studio?

- ◯ **Utilizzo del tablet per la raccolta dati [1]**
- ◯ **Altro [2] ________________________**

Durante la visita di valutazione al basale, al termine della compilazione dei 3 questionari, si chiede al paziente di rispondere alle seguenti domande:

Come giudica l'uso del tablet computer per rispondere alle domande?

- ◯ **Molto difficile [1]**
- ◯ **Un po' difficile [2]**
- ◯ **Né difficile né facile [3]**
- ◯ **Un po' facile [4]**
- ◯ **Molto facile [5]**

Quanto è soddisfatto per l'utilizzo del tablet per rilevare la sua qualità della vita?

- ◯ **Molto insoddisfatto [1]**
- ◯ **Piuttosto insoddisfatto [2]**
- ◯ **Né soddisfatto né insoddisfatto [3]**
- ◯ **Soddisfatto [4]**
- ◯ **Molto soddisfatto [5]**

ATTACHMENT V - INFORMATION SHEET AND CONSENT FORM

STUDIO PRINCIPALE

Titolo dello studio: *Trial clinico controllato e randomizzato, con disegno a cluster stepped-wedge, per valutare una strategia volta ad ottimizzare gli outcomes psicosociali in pazienti affetti da cancro*

FOGLIO INFORMATIVO PER IL PAZIENTE

Gentile Signora/e,
le stiamo chiedendo di partecipare ad uno studio clinico. Prima che Lei decida, è importante che abbia tutte le informazioni sul perché questo studio viene fatto e che cosa comporta. Si prenda tutto il tempo necessario per leggere queste informazioni attentamente. Chieda ai medici che l'hanno in cura qualsiasi spiegazione su aspetti che non le sono chiari. Se qualche termine non è comprensibile può guardare la sezione "Cosa vuol dire".

Lo studio coinvolge 15 oncologie italiane e si propone di valutare l'efficacia di una strategia volta a favorire le cure psicosociali del paziente affetto da tumore. Per la realizzazione dello studio è necessaria la collaborazione e la disponibilità di persone a cui, come Lei, è stato di recente diagnosticato un tumore e stanno per iniziare un nuovo trattamento terapeutico.
Questo foglio riporta le informazioni principali sulle finalità e le modalità di conduzione dello studio a cui Le viene chiesto di partecipare. Le viene consegnato in anticipo al fine di poter leggere tranquillamente le informazioni e discuterle con i Suoi familiari e il Suo medico; nel caso decidesse di partecipare, Le verrà chiesto di firmare l'allegato Modulo di Consenso Informato che indica la sua accettazione a partecipare allo studio.

Quale è lo scopo dello studio?
La malattia oncologica ha un forte impatto sulla vita dei pazienti e dei loro familiari, che non si limita alla sintomatologia e agli effetti collaterali delle terapie. La ricerca evidenzia infatti l'esistenza di una vasta gamma di bisogni psicosociali, definiti come aspetti psicologici, emozionali, sociali e spirituali della salute, che spesso non vengono rilevati o adeguatamente affrontati. Nonostante l'esistenza di numerose Linee Guida (LG) che raccomandano interventi per l'assistenza psicosociale in oncologia, molti pazienti che trarrebbero beneficio da questi interventi, in realtà non li ricevono.
Questa consapevolezza ha ispirato uno studio nazionale denominato HuCare, finanziato dal Ministero della Salute e dalla Regione Lombardia, concluso nel 2014, che ha dimostrato la fattibilità di una strategia, Hucare Quality Improvement Strategy (HQIS), che favorisce l'applicazione nei reparti di oncologia di interventi psicosociali, raccomandati da LG internazionali. La strategia prevede la formazione dello staff clinico sulle tecniche di comunicazione e il supporto di un team di esperti, che aiuta lo staff a comprendere gli ostacoli, identificare soluzioni e rafforzare la motivazione al cambiamento, cioè a prestare maggiore attenzione ai bisogni e ad offrire le cure psicosociali necessarie ai pazienti che iniziano una nuova terapia.
Questo studio ha come obiettivo principale quello di valutare se l'introduzione della strategia HQIS nei reparti di oncologia migliora la qualità della vita dei pazienti con cancro.

Quali sono le caratteristiche di questo studio?
La metodologia di conduzione dello studio prevede la randomizzazione dei centri di oncologia partecipanti, cioè l'assegnazione casuale della strategia HQIS ad ogni centro oncologico in tempi diversi, stabiliti mediante un programma informatico. Tale modalità consente che tutti i centri applichino gli interventi psicosociali, anche se in periodi diversi, e che sia possibile la misura e il confronto dell'impatto di questa strategia sui pazienti, prima e dopo la sua applicazione nel centro.
Inoltre, al fine di consentire un confronto reale e indipendente, cioè non influenzato da fattori che potrebbero confondere i risultati dello studio, Lei non sarà informato se al momento della raccolta dei dati è in atto la strategia nel centro o se deve essere ancora implementata. Tale modalità viene denominata "in cieco", cioè non nota al paziente.
Lo studio prevede due momenti di raccolta dati, uno prima dell'inizio del primo ciclo di terapia e uno dopo circa tre mesi dall'arruolamento in studio, durante la visita di follow-up – cioè di verifica del Suo stato di salute da parte dell'oncologo del centro.

Perché lo studio è stato proposto a me?
Le stiamo proponendo di partecipare a questo studio perché Lei ha una malattia tumorale e sta per iniziare un trattamento medico che prevede la somministrazione di farmaci. Questa fase è molto delicata ed è generalmente quella in cui viene rilevato un peggioramento della qualità di vita dei pazienti e la necessità di cure psicosociali oltre che farmacologiche.
E' previsto che partecipino a questo studio circa 720 pazienti curati in 15 Istituti e Ospedali italiani. Il protocollo di questo studio è stato approvato dal Comitato Etico della struttura da cui Lei è in cura, un organismo indipendente che ha il compito di tutelare l'interesse del paziente che partecipa ad uno studio clinico.

Cosa comporta la mia partecipazione?
Se Lei deciderà di partecipare allo studio Le verrà chiesto di compilare 3 questionari tramite tablet, computer di dimensioni ridotte, costituito da una tavoletta sulla quale si scrive utilizzando un apposito stilo. E' prevista la rilevazione delle informazioni in due momenti diversi, prima di iniziare il primo ciclo di terapia e dopo circa tre mesi; generalmente non occorrono più di 20 minuti per la compilazione dei questionari, in quanto Le viene richiesto di indicare una delle risposte già elencate. Durante la rilevazione sarà assistito da un infermiere addestrato allo scopo, che le spiegherà come utilizzare il tablet e potrà rispondere ad eventuali chiarimenti. Sarà importante rispondere a tutte le domande dei questionari, al fine di consentire alla fine dello studio di poter affermare se la strategia adottata nelle oncologie consente di ottenere benefici per i pazienti in termini di miglioramento della qualità di vita.
La partecipazione allo studio prevede che il Suo medico trasmetta i dati relativi alle Sue caratteristiche cliniche e demografiche alla UO Ricerca e Innovazione dell'Azienda Ospedaliero-Universitaria di Parma, per l'elaborazione dei risultati. Tutti i dati saranno elaborati in forma anonima: solo il Suo medico sarà in grado di legare il Suo nominativo alla Sua situazione clinica registrata nell'archivio elettronico dello studio.
Solo i pazienti che hanno accettato e firmato il consenso informato partecipano allo studio e il paziente che inizialmente accetta di partecipare può comunque ritirare il suo consenso in ogni momento senza dover dare alcuna spiegazione.

Quali sono i vantaggi e gli svantaggi a partecipare allo studio?
La partecipazione non comporterà per Lei nessuna modifica dei trattamenti comunemente

somministrati.
La Sua partecipazione a questo studio permetterà di ottenere informazioni scientifiche che potranno in futuro migliorare il trattamento dei pazienti con cancro.

Chi promuove lo studio?
Il presente studio è promosso e sostenuto economicamente dall'Associazione Italiana di Oncologia Medica (www.aiom.it) e dall'Associazione di volontariato MedeA ((www.medeacremona.it), ed è coordinato dall'UO Oncologia di Cremona. L'AIOM è un'associazione scientifica il cui scopo principale è promuovere la ricerca sperimentale e clinica, la prevenzione primaria e secondaria (screening e diagnosi precoce e tempestiva), la qualità delle cure e la continuità terapeutica del paziente oncologico, e di contribuire alla formazione professionale di oncologi medici e operatori sanitari.

Garanzie a tutela del paziente partecipante allo studio
La partecipazione dei pazienti allo studio è totalmente libera. Questo significa che essi possono liberamente decidere di non partecipare allo studio, e dunque di non firmare il consenso, senza compromettere l'assistenza che riceveranno in seguito dal proprio medico. Inoltre, i pazienti che inizialmente accettano di partecipare allo studio possono in seguito ritirare in ogni momento il loro consenso senza dover dare alcuna spiegazione. Lei non avrà nessun costo da sostenere per poter partecipare allo studio.

Assicurazione
Poiché lo studio prevede l'implementazione di una strategia volta a favorire interventi psicosociali EBM, attraverso la formazione dei professionisti e la riorganizzazione delle attività, senza alcuna modifica dell'iter diagnostico-terapeutico dei pazienti, tutti i partecipanti allo studio sono coperti dalla assicurazione prevista dall'Ospedale per i propri pazienti, in accordo alle leggi e alle norme vigenti.

Confidenzialità delle informazioni e tutela della privacy
Quando il paziente firma il proprio consenso a partecipare allo studio, esprime anche il suo consenso al fatto che informazioni sanitarie che lo riguardano vengano utilizzate per gli scopi suddetti e vengano visionate da tutti coloro che sono coinvolti nell'effettuazione dello studio (personale sanitario, personale che elabora i dati, personale ispettivo e quant'altri abilitati dal protocollo di studio e/o dalle normative vigenti).
Tutte le informazioni (personali, cliniche) raccolte durante questo studio sono confidenziali e verranno trattati nel rispetto della normativa vigente, ai sensi del del D.L. n° 196 del 30 giugno 2003 in materia di diritto alla riservatezza dei dati personali e della Deliberazione n° 52 del 24 luglio 2008 in materia delle linee guida per i trattamenti di dati personali nell'ambito delle sperimentazioni cliniche.
Tutte le informazioni saranno pubblicate solo in forma anonima e nel loro complesso, evitando accuratamente e rigorosamente qualunque dettaglio che possa in qualche modo consentire a terzi di risalire all'identità del paziente.
Il titolare del trattamento dei dati è l'Azienda Ospedaliero-Universitaria di Parma. Per ogni necessità o domanda, Lei potrà inoltre rivolgersi al Suo medico, che avrà cura di fornirLe ogni ulteriore chiarimento in merito.
Una copia di questo Modulo Informativo e una copia dell'eventuale Consenso Informato restano in possesso del paziente che accetti di partecipare allo studio.

Responsabile
Il Responsabile scientifico dello studio è il Dottor Rodolfo Passalacqua, UO Oncologia dell'Azienda Socio-Sanitaria Territoriale di Cremona.

"Cosa vuol dire":
Linee guida (LG)
Le linee guida sono "raccomandazioni di comportamento clinico, elaborate mediante un processo di revisione sistematica della letteratura e delle opinioni di esperti, con lo scopo di aiutare i medici e i pazienti a decidere le modalità assistenziali più appropriate in specifiche situazioni cliniche".

Randomizzazione
Processo di assegnazione casuale dei partecipanti a uno dei gruppi previsti dallo studio. La randomizzazione ha lo scopo di rendere simili i gruppi per le loro caratteristiche. Questo permette l'applicabilità dei modelli probabilistici sui quali si regge dal punto di vista metodologico l'intero studio.

Follow-up (FU)
Visita di controllo con il medico, periodica e programmata, durante la quale viene verificato lo stato di salute del paziente e confrontato con quanto rilevato nella visita precedentemente realizzata. Lo scopo è comprendere se sono necessari ulteriori approfondimenti diagnostici o modifiche nelle cure in atto.

Hucare Quality Improvement Strategy (HQIS)
E' una strategia volta a favorire l'applicazione nei reparti di oncologia di interventi volti a migliorare lo stato psicosociale dei pazienti con cancro. E' stata ideata e applicata in 28 oncologie italiane nell'ambito del progetto Hucare (Humanization in cancer care), finanziato dal Ministero della Salute e dalla Regione Lombardia.

CONSENSO INFORMATO ALLA PARTECIPAZIONE ALLO STUDIO

Questo modulo, deve essere firmato da Lei solo nel caso decida di partecipare allo studio clinico generale. E' importante che Lei abbia discusso approfonditamente con il Medico prima di firmare questo consenso, anche sulla base del foglio informativo a cui esso si riferisce.

Partecipano allo studio solo i Pazienti che accettano. Il Paziente può ritirare il suo consenso in ogni momento.

Io sottoscritto/a, ________________________________nato/a a _______________________

(prov. ____) il ___/___/_____ residente in ____

(prov. ____) nella mia piena capacità di intendere e di volere:

- Confermo di essere stato/a dovutamente informato/a circa lo studio e aver avuto tempo sufficiente per chiedere ulteriori informazioni e per considerare la mia partecipazione ad esso.
- Ho ricevuto una copia del Foglio Informativo per il paziente.
- Tutti i miei diritti mi sono stati spiegati chiaramente.
- Sono consapevole che la partecipazione a questo studio è del tutto volontaria e che sarò libero/a di accettare o rifiutare di prenderne parte, come pure di ritirare il mio consenso in un secondo momento, senza la necessità di motivare la mia decisione e senza influenzare, per questo, la qualità e l'adeguatezza delle successive decisioni terapeutiche sulla mia persona.
- Sono consapevole e acconsento che tutte le informazioni (personali, cliniche) raccolte durante lo studio siano considerate confidenziali e vengano trattate nel rispetto della normativa vigente ai sensi del D.L. n° 196 del 30 giugno 2003 in materia di diritto alla riservatezza dei dati personali e della Deliberazione n° 52 del 24 luglio 2008 in materia delle linee guida per i trattamenti di dati personali nell'ambito delle sperimentazioni cliniche di medicinali
- Acconsento a partecipare allo studio clinico suddetto

Nome del Paziente: ______________________________

Firma del Paziente: _____________________________ Data: ________________

Nome del Medico: ______________________________

Firma del Medico: ______________________________ Data: ________________

STUDIO DI FATTIBILITA'

Titolo dello studio: *Indagine pilota per misurare il gradimento dei pazienti e la fattibilità dell'utilizzo di un dispositivo informatico (tablet) per la misura della Qualità di Vita e dei bisogni psicosociali*

FOGLIO INFORMATIVO PER IL PAZIENTE

Gentile Signora/e,
le stiamo chiedendo di partecipare ad uno studio clinico. Prima che Lei decida, è importante che abbia tutte le informazioni sul perché questo studio viene fatto e che cosa comporta. Si prenda tutto il tempo necessario per leggere queste informazioni attentamente. Chieda ai medici che l'hanno in cura qualsiasi spiegazione su aspetti che non le sono chiari.

L'indagine viene condotta presso l'Oncologia di Cremona, centro di coordinamento di uno studio nazionale che coinvolge 15 oncologie italiane e che si propone di valutare l'efficacia di una strategia volta a favorire le cure psicosociali del paziente affetto da tumore. Per la realizzazione dello studio nazionale è necessario condurre questa indagine limitata alla oncologia di Cremona; a tale scopo, viene chiesta la collaborazione e la disponibilità di circa 20 pazienti a cui, come Lei, è stato di recente diagnosticato un tumore e stanno per iniziare un nuovo trattamento terapeutico.
Questo foglio riporta le informazioni principali sulle finalità e le modalità di conduzione dello studio a cui Le viene chiesto di partecipare. Le viene consegnato in anticipo al fine di poter leggere tranquillamente le informazioni e discuterle con i Suoi familiari e il Suo medico; nel caso decidesse di partecipare, Le verrà chiesto di firmare l'allegato Modulo di Consenso Informato che indica la sua accettazione a partecipare allo studio.

Quale è lo scopo dello studio?
La malattia oncologica ha un forte impatto sulla vita dei pazienti e dei loro familiari, che non si limita alla sintomatologia e agli effetti collaterali delle terapie. La ricerca evidenzia infatti l'esistenza di una vasta gamma di bisogni psicosociali, definiti come aspetti psicologici, emozionali, sociali e spirituali della salute, che spesso non vengono rilevati o adeguatamente affrontati.
Di recente vengono condotti numerosi studi in cui vengono misurati, mediante questionari validati a livello internazionale, la qualità della vita dei pazienti e i loro bisogni, per poter fornire non solo le terapie farmacologiche ma anche le cure psicosociali.
Questo studio ha come obiettivo principale quello di raccogliere alcune informazioni relative ai tempi necessari per la compilazione di tre questionari e al gradimento dei pazienti in merito all'uso di dispositivi elettronici per la raccolta dei dati.

Quali sono le caratteristiche di questo studio?
Lo studio prevede solo una raccolta di dati che saranno analizzati e utilizzati per pianificare meglio le attività di uno studio più ampio condotto a livello nazionale su circa 720 pazienti che, come lei, hanno una malattia tumorale e stanno per iniziare un trattamento medico che prevede la somministrazione di farmaci.
Il protocollo di questo studio è stato approvato dal Comitato Etico di Cremona, un organismo indipendente che ha il compito di tutelare l'interesse del paziente che partecipa ad uno studio clinico.

Cosa comporta la mia partecipazione?
Se Lei deciderà di partecipare allo studio Le verrà chiesto di compilare 3 questionari tramite tablet, computer di dimensioni ridotte, costituito da una tavoletta sulla quale si scrive utilizzando il dito. E' prevista la rilevazione prima di iniziare il primo ciclo di terapia e generalmente non occorrono più di 20 minuti per la compilazione dei questionari, in quanto Le viene richiesto di indicare una delle risposte già elencate. Durante la rilevazione sarà assistito da un infermiere addestrato allo scopo, che le spiegherà come utilizzare il tablet e potrà rispondere ad eventuali chiarimenti. Sarà importante rispondere a tutte le domande dei questionari, al fine di consentire alla fine dello studio di poter pianificare correttamente lo studio nazionale.
La partecipazione allo studio prevede che il Suo medico trasmetta i dati relativi alle Sue caratteristiche cliniche e demografiche alla UO Ricerca e Innovazione dell'Azienda Ospedaliero-Universitaria di Parma, per l'eleborazione dei risultati. Tutti i dati saranno elaborati in forma anonima: solo il Suo medico sarà in grado di legare il Suo nominativo alla Sua situazione clinica registrata nell'archivio elettronico dello studio.
Solo i pazienti che hanno accettato e firmato il consenso informato partecipano allo studio e il paziente che inizialmente accetta di partecipare può comunque ritirare il suo consenso in ogni momento senza dover dare alcuna spiegazione.

Quali sono i vantaggi e gli svantaggi a partecipare allo studio?
La partecipazione non comporterà per Lei nessuna modifica dei trattamenti comunemente somministrati ne nuove conoscenze specifiche sul Suo stato di salute.
La Sua partecipazione a questo studio permetterà di ottenere informazioni scientifiche che potranno in futuro migliorare il trattamento dei pazienti con cancro.

Chi promuove lo studio?
Il presente studio è sostenuto economicamente dall'Associazione Italiana di Oncologia Medica (www.aiom.it) e dall'Associazione di volontariato MedeA ((www.medeacremona.it), e coordinato dall'UO Oncologia di Cremona. L'AIOM è un'associazione scientifica il cui scopo principale è promuovere la ricerca sperimentale e clinica, la prevenzione primaria e secondaria (screening e diagnosi precoce e tempestiva), la qualità delle cure e la continuità terapeutica del paziente oncologico, e di contribuire alla formazione professionale di oncologi medici e operatori sanitari.

Garanzie a tutela del paziente partecipante allo studio

La partecipazione dei pazienti allo studio è totalmente libera. Questo significa che essi possono liberamente decidere di non partecipare allo studio, e dunque di non firmare il consenso, senza compromettere l'assistenza che riceveranno in seguito dal proprio medico. Inoltre, i pazienti che inizialmente accettano di partecipare allo studio possono in seguito ritirare in ogni momento il loro consenso senza dover dare alcuna spiegazione. Lei non avrà nessun costo da sostenere per poter partecipare allo studio.

Assicurazione

Non prevedendo alcun trattamento farmacologico sperimentale, tutti i partecipanti allo studio sono coperti dalla assicurazione dell'Ospedale prevista per tutti i pazienti, che copre anche i rischi inerenti la partecipazione dello studio in accordo alle leggi e alle norme vigenti.

Confidenzialità delle informazioni e tutela della privacy

Quando il paziente firma il proprio consenso a partecipare allo studio, esprime anche il suo consenso al fatto che informazioni sanitarie che lo riguardano vengano utilizzate per gli scopi suddetti e vengano visionate da tutti coloro che sono coinvolti nell'effettuazione dello studio (personale sanitario, personale che elabora i dati, personale ispettivo e quant'altri abilitati dal protocollo di studio e/o dalle normative vigenti).

Tutte le informazioni (personali, cliniche) raccolte durante questo studio sono confidenziali e verranno trattati nel rispetto della normativa vigente, ai sensi del del D.L. n° 196 del 30 giugno 2003 in materia di diritto alla riservatezza dei dati personali e della Deliberazione n° 52 del 24 luglio 2008 in materia delle linee guida per i trattamenti di dati personali nell'ambito delle sperimentazioni cliniche.

Tutte le informazioni saranno pubblicate solo in forma anonima e nel loro complesso, evitando accuratamente e rigorosamente qualunque dettaglio che possa in qualche modo consentire a terzi di risalire all'identità del paziente.

Il titolare del trattamento dei dati è l'Azienda Ospedaliero-Universitaria di Parma. Per ogni necessità o domanda, Lei potrà inoltre rivolgersi al Suo medico, che avrà cura di fornirLe ogni ulteriore chiarimento in merito.

Una copia di questo Modulo Informativo e una copia dell'eventuale Consenso Informato restano in possesso del paziente che accetti di partecipare allo studio.

Responsabile

Il Responsabile scientifico dello studio a livello locale è il Dottor Rodolfo Passalacqua, UO Oncologia dell'Azienda Socio-Sanitaria Territoriale di Cremona

ATTACHMENT VI - *LETTER FOR GENERAL PRACTITIONER*

Titolo dello studio: *Trial clinico controllato e randomizzato, con disegno a cluster stepped-wedge, per valutare una strategia volta ad ottimizzare gli outcomes psicosociali in pazienti affetti da cancro*

Gentile Collega,

con la presente vogliamo informarti che il Sig./la Sig.ra ……………………………………………
è stato/a incluso/a, dopo aver ottenuto il consenso, nello studio prospettico, multicentrico HuCare2 (Humanization in Cancer Care) che coinvolge 16 oncologie italiane e si propone di valutare l'efficacia di una strategia volta a favorire le cure psicosociali del paziente affetto da tumore.
Di seguito riportiamo brevemente le informazioni principali sulle finalità e le modalità di conduzione dello studio.

Quale è lo scopo dello studio?
La malattia oncologica ha un forte impatto sulla vita dei pazienti e dei loro familiari, che non si limita alla sintomatologia e agli effetti collaterali delle terapie. La ricerca evidenzia infatti l'esistenza di una vasta gamma di bisogni psicosociali, definiti come aspetti psicologici, emozionali, sociali e spirituali della salute, che spesso non vengono rilevati o adeguatamente affrontati. Nonostante l'esistenza di numerose Linee Guida (LG) che raccomandano interventi per l'assistenza psicosociale in oncologia, molti pazienti che trarrebbero beneficio da questi interventi, in realtà non li ricevono.
Questa consapevolezza ha ispirato uno studio nazionale denominato HuCare, finanziato dal Ministero della Salute e dalla Regione Lombardia, concluso nel 2014, che ha dimostrato la fattibilità di una strategia, Hucare Quality Improvement Strategy (HQIS), che favorisce l'applicazione nei reparti di oncologia di interventi psicosociali, raccomandati da LG internazionali. La strategia prevede la formazione dello staff clinico sulle tecniche di comunicazione e il supporto di un team di esperti, che aiuta lo staff a comprendere gli ostacoli, identificare soluzioni e rafforzare la motivazione al cambiamento, cioè a prestare maggiore attenzione ai bisogni e ad offrire le cure psicosociali necessarie ai pazienti che iniziano una nuova terapia.
Questo studio ha come obiettivo principale quello di valutare se l'introduzione della strategia HQIS nei reparti di oncologia migliora la qualità della vita dei pazienti con cancro.

Quali sono le caratteristiche di questo studio?
La metodologia di conduzione prevede la realizzazione di uno studio di fattibilità presso l'Oncologia di Cremona, centro di coordinamento, e successivamente di uno studio sperimentale presso altre 15 oncologie italiane, randomizzate ad applicare nel proprio contesto

la strategia HQIS in tempi diversi (disegno dello studio stepped-wedge). Tale metodologia consente che tutti i centri applichino gli interventi psicosociali, anche se in periodi diversi, e che sia possibile la misura e il confronto dell'impatto di questa strategia sui pazienti, prima e dopo la sua applicazione nel centro.
Lo studio di fattibilità (condotto solo presso l'Oncologia di Cremona) prevede un'unica rilevazione volta a raccogliere alcune informazioni relative ai tempi necessari per la compilazione di tre questionari e al gradimento dei pazienti in merito all'uso di dispositivi elettronici per la raccolta dei dati.
Lo studio principale prevede due momenti di raccolta dati, uno prima dell'inizio del primo ciclo di terapia e uno dopo circa tre mesi dall'arruolamento in studio, durante la visita di follow-up.
La partecipazione non comporterà per il paziente nessuna modifica dei trattamenti farmacologici comunemente somministrati.
Il protocollo dello studio è stato approvato dal Comitato Etico.

Pazienti inclusi
E' previsto che partecipino a questo studio circa 20 pazienti arruolati nel Centro di coordinamento di Cremona e 720 pazienti curati in 15 Istituti e Ospedali italiani.
Saranno arruolati pazienti con cancro, di qualunque tipo e stadio, che in modo consecutivo accedono alle strutture partecipanti (DH e ambulatori) in un periodo indice e che rispondono ai seguenti
Criteri di inclusione

- Età > 18 e < 75 anni
- Diagnosi (istologica o citologica) di tumore solido da non più di un mese
- Che devono iniziare un primo trattamento medico: chemioterapia EV, farmaci orali, farmaci a target molecolare
- Sopravvivenza attesa > 3 mesi
- Buona comprensione della lingua italiana
- Che hanno letto, compreso e sottoscritto il consenso informato

Criteri di esclusione

- Pregressa chemioterapia o altro trattamento medico per tumore
- Arruolato nel medesimo trial in un periodo precedente
- Partecipazione in atto ad altri trial che prevedono la misura di PRO
- In trattamento da uno psicologo o psichiatra o con antidepressivi
- Ricoverato in degenza ordinaria
- Presenza di condizioni patologiche mentali o psichiatriche, dovute al tumore o a patologie concomitanti, che interferiscono con lo stato di coscienza o con la capacità di giudizio
- Impossibilità a completare il questionario o a garantire il follow-up a tre mesi

Chi promuove lo studio?
Il presente studio è sostenuto economicamente dall'Associazione Italiana di Oncologia Medica (www.aiom.it) e dall'Associazione di volontariato MedeA ((www.medeacremona.it)

Responsabile
Il Responsabile scientifico e coordinatore principale dello studio è il Dottor Rodolfo Passalacqua, UO Oncologia dell'Azienda Socio-Sanitaria Territoriale di Cremona.

Se desideri ulteriori informazioni sullo studio non esitare a contattare il Dr. ______________

____, responsabile dello studio HuCare2

per il Centro di ____

Cordialmente,

Firma _________________________________

ATTACHMENT VII – GANTT DIAGRAM

Anno 2016

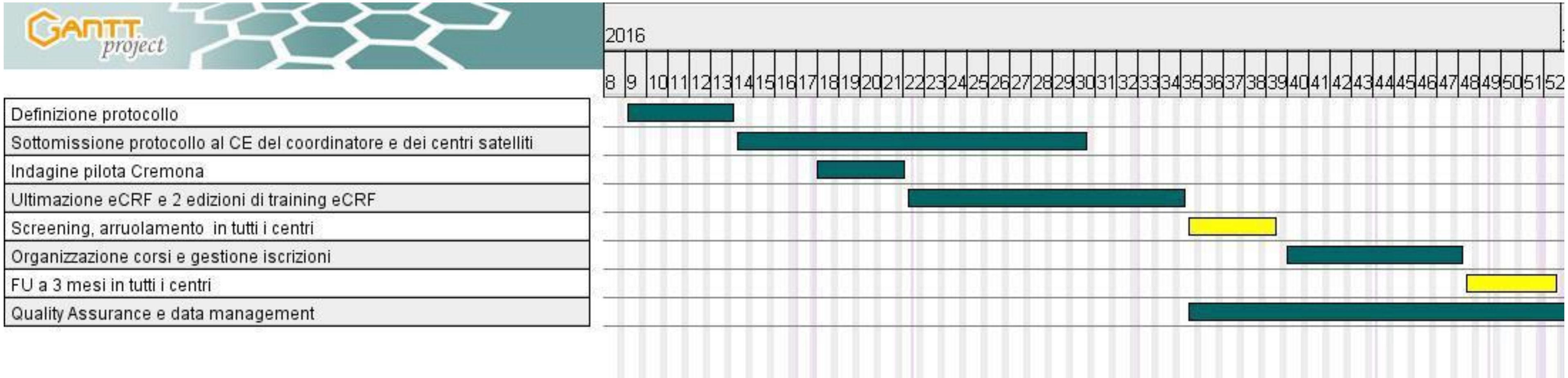

Anno 2017

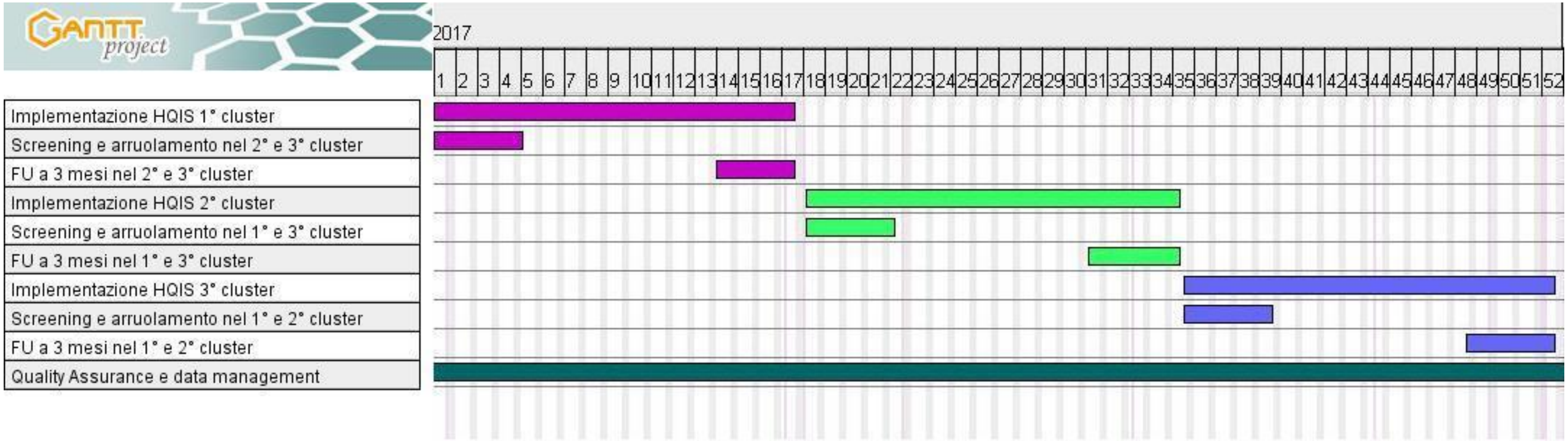

Anno 2018

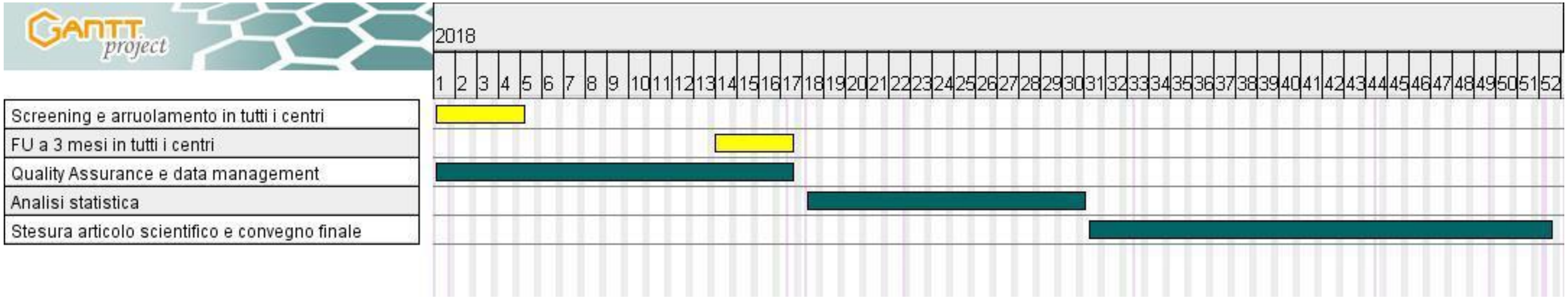

ATTACHMENT VIII – LIST OF PARTICIPATING CENTERS

	Direttore UO	Istituzione	Città
	Rodolfo Passalacqua	ASST Istituti Ospitalieri	CREMONA
1	Mario Airoldi	Azienda Ospedaliero Universitaria Città della Salute e della Scienza	TORINO
2	Giuseppe Altavilla	Azienda Ospedaliera Universitaria Policlinico Universitario "G. Martino"	MESSINA
3	Liberato Di Lullo	Azienda Ospedaliera Molise - Presidio Ospedaliero Cardarelli	CAMPOBASSO
4	Gianpiero Fasola	Azienda Ospedaliero Universitaria	UDINE
5	Giuseppe Procopio	IRCCS Istituto tumori	MILANO
6	Stefania Gori	Ospedale Sacro Cuore "Don Calabria" Presidio ospedaliero accreditato	VERONA
7	Saverio Cinieri	Azienda USL – Presidio "Di Summa - Perrino"	BRINDISI
8	Rodolfo Mattioli	Azienda Ospedaliera Ospedali Riuniti Marche Nord	FANO
9	Vincenzo Monesarchio	Azienda Ospedaliera dei Colli	NAPOLI
10	Salvatore Palazzo	Azienda Ospedaliera	COSENZA
11	Antonio Pazzola	ASL Ospedale SS. Annunziata	SASSARI
12	Antonio Russo	Azienda Ospedaliera "P. Giaccone"	PALERMO
13	Maria Giuseppa Sarobba	Azienda Sanitaria Locale	NUORO
14	Giorgio Scagliotti	Azienda Ospedaliero Universitaria "San Luigi Gonzaga"	TORINO
15	Filippo Zerilli	Azienda Ospedaliera Sanitaria Provinciale	TRAPANI
