# Supplementary material for: Effectiveness of a Psychosocial Care Quality Improvement Strategy to Address Quality of Life in Patients With Cancer: The HuCare2 Stepped-Wedge Cluster Randomized Trial
Source: JAMA Netw Open. 2021 Oct 14;4(10):e2128667. doi: 10.1001/jamanetworkopen.2021.28667 (PMC8517739; doi:10.1001/jamanetworkopen.2021.28667)
Supplement: Supplement 2. — eMethods. Instruments Used by the Improvement Team eTable 1. Percentage of Patients Who Exhibit Change in the Emotional or Social Functions of HRQoL eTable 2. Long-term Effect of the HQIS on the Emotional and Social Functions of HRQoL eTable 3. Effects of HQIS on Global HRQoL and Other Functions and Symptoms eTable 4. Effect of HQIS on Mood Disorder Measured With the HADS-D eTable 5. Results of the Subgroup Analysis Aimed to Identify Any Effect Modifiers on the Emotional Function of HRQoL [file jamanetwopen-e2128667-s002.pdf]

Supplementary Online Content

Caminiti C, Annunziata MA, Verusio C, et al. Effectiveness of a psychosocial care quality improvement strategy to address quality of life in patients with cancer: the HuCare2 stepped-wedge cluster randomized trial. *JAMA Netw Open*. 2021;4(10):e2128667. doi:10.1001/jamanetworkopen.2021.28667

eMethods. Instruments Used by the Improvement Team

eTable 1. Percentage of Patients Who Exhibit Change in the Emotional or Social Functions of HRQoL

eTable 2. Long-term Effect of the HQIS on the Emotional and Social Functions of HRQoL

eTable 3. Effects of HQIS on Global HRQoL and Other Functions and Symptoms

eTable 4. Effect of HQIS on Mood Disorder Measured With the HADS-D

eTable 5. Results of the Subgroup Analysis Aimed to Identify Any Effect Modifiers on the Emotional Function of HRQoL

This supplementary material has been provided by the authors to give readers additional information about their work.

© 2021 Caminiti C et al. *JAMA Network Open.*

eMethods. instruments used by the Improvement Team

The HQIS followed the Plan-Do-Study-Act (PDSA) cycle, which uses small tests of change to optimize a process24. In this view, the problems and solutions identified during one site visit performed by the IT were used to introduce changes, and to inform the following visit. To this end, information for center support activities was systematically collected at different time points using standardized instruments specifically created for the project. This enabled to timely identify barriers and facilitators of implementation in different settings as the project unfolded, and ensured uniformity of action.

Tools for context analysis

At the start-up visit, performed by the sociologist and the psychologist of the IT, information on each staff member's motivation and opinion about the project was collected using a 6-item questionnaire where answers were given on a Likert scale. Participants were also asked to write anonymously on cards what they felt was the main strength and weakness of the project. These cards were then read aloud and used to inspire discussion on barriers and solutions.

After each start-up visit, a report describing the main observations on context analysis was provided to all IT members, to help prepare for the next on-site visit at the center.

Trend Template

To standardize the collection of information, and to obtain data which could be easily analyzed and acted upon, information gathered during the start-up visit was also entered into a "trend template". This tool included a grid where, for each of the 5 recommendations, the IT members reported difficulties that had emerged and possible solutions identified to tackle them. A trend template was created for each center. For every subsequent visit, rows were added to the grid so that updates could be noted, still retaining information entered at previous visits. This enabled IT members to verify if issues had been resolved, and guided further debate with staff when problems persisted. The Template also included a section where IT members provided a descriptive, qualitative summary of the visit.

© 2021 Caminiti C et al. *JAMA Network Open.*

eTable 1. Percentage of patients who exhibit change in the emotional or social functions of HRQoL

**EMOTIONAL**	**HQIS**		**Usual Care**		***Total***	
**T1 vs T0**	(n=332)		(n=315)		*(n=647)*	
Improved	151	(45.4)	117	(37)	*267*	(41.3)
Unchanged	64	(19.4)	68	(21.7)	*133*	(20.5)
Worsened	117	(35.2)	130	(41.3)	*247*	(38.2)
**SOCIAL**	**HQIS**		**Usual Care**		***Total***	
**T1 vs T0**	(n=332)		(n=315)		*(n=647)*	
Improved	74	(22.2)	63	(19.9)	*136*	(21.1)
Unchanged	135	(40.6)	146	(46.4)	*281*	(43.4)
Worsened	123	(37.1)	106	(33.7)	*230*	(35.5)

© 2021 Caminiti C et al. *JAMA Network Open.*

eTable 2. Long term effect of the HQIS on the emotional and social functions of HRQoL. Regression coefficient (β) , Odds Ratio (OR) and 95% confidence intervals (CI) of the variables included in the analysis, and P-value of the Binomial Beta Regression Model.

	**EMOTIONAL FUNCTION**					**SOCIAL FUNCTION**				
**Variable**	**β**	**OR**	**95%CI**		**P-value**	**β**	**OR**	**95%CI**		**P-value**
Intercept	*-0.192*	*0.825*	*0.596*	*1.055*	*0.102*	*-0.157*	*0.855*	*0.704*	*1.005*	*0.041*
Post Intervention Epoch (PIE)	***-0.046***	***0.955***	***0.911***	*0.999*	***0.043***	***0.057***	***1.059***	***0.867***	*1.251*	***0.560***
HQIS Implementation Epoch 1st vs 3rd (Cluster 1)	*0.017*	*1.017*	*0.871*	*1.163*	*0.821*	*-0.147*	*0.863*	*0.702*	*1.024*	*0.074*
HQIS Implementation Epoch 2nd vs 3rd (Cluster 2)	*0.132*	*1.141*	*1.017*	*1.265*	*0.039*	*0.071*	*1.074*	*0.936*	*1.212*	*0.313*
INTERACTION: PIE x Cluster 1	*0.049*	*1.050*	*1.003*	*1.097*	*0.044*	*0.020*	*1.020*	*0.814*	*1.226*	*0.848*
INTERACTION: PIE x Cluster 2	*0.029*	*1.030*	*0.982*	*1.078*	*0.230*	*-0.052*	*0.950*	*0.741*	*1.159*	*0.628*
Age (yr)	*-0.004*	*0.996*	0.993	0.999	*0.018*	--	--	--	--	--
Sex (male)	0.057	1.059	0.970	1.148	0.206	--	--	--	--	--
Married	--	--	--	--	--	0.099	1.104	0.994	1.214	0.078
Instruction (more primary)	--	--	--	--	--	--	--	--	--	--
No Metastases	--	--	--	--	--	--	--	--	--	--
Other than chemotherapy	--	--	--	--	--	0.140	1.150	1.017	1.283	0.039
ECOG PS	--	--	--	--	--	--	--	--	--	--
Needs met	0.174	1.191	1.109	1.272	<0.001	0.117	1.124	1.024	1.224	0.023
**Log-phi (SE)**	**-2.793 (0.065)**					**-2.518 (0.063)**				

© 2021 Caminiti C et al. *JAMA Network Open.*

eTable 3. Effects of HQIS on global HRQoL and other functions and symptoms. Regression coefficient (β), Odds Ratio (OR) and 95% confidence intervals (CI) of the variables included in the analysis, and P-value of the Binomial Beta Regression Model.

Variable	GLOBAL HRQoL: Univariate analysis P-value	Multivariate analysis β	OR	95%CI		P-value
Intercept		-0.225	0.799	0.606	0.991	0.022
HQIS vs Usual Care		0.012	1.012	0.909	1.115	0.823
HQIS Implementation Epoch 1st vs 3rd		0.021	1.021	0.887	1.155	0.760
HQIS Implementation Epoch 2nd vs 3rd		-0.004	0.996	0.877	1.116	0.951
Age (yr)	0.607	--	--	--	--	--
Sex (male)	0.763	--	--	--	--	--
Married	0.081	0.104	1.110	0.993	1.226	0.082
Instruction (more primary)	0.548	--	--			--
No Metastases	0.004	-0.146	0.865	0.758	0.971	0.008
Other than chemotherapy	0.274	--	--	--	--	--
ECOG PS	0.208	--	--	--	--	--
Needs met	0.007	0.157	1.170	1.063	1.277	0.004
**Log-phi (SE)**		**-2.376 (0.061)**				

Variable	PHYSICAL Function: Univariate analysis P-value	Multivariate analysis β	OR	95%CI		P-value
Intercept		-0.187	0.830	0.693	0.966	0.007
HQIS vs Usual Care		0.065	1.067	0.967	1.168	0.203
HQIS Implementation Epoch 1st vs 3rd		0.025	1.025	0.897	1.154	0.706
HQIS Implementation Epoch 2nd vs 3rd		0.044	1.045	0.930	1.159	0.453
Age (yr)	0.189	--	--	--	--	--
Sex (male)	0.806	--	--	--	--	--
Married	0.650	--	--	--	--	--
Instruction (more primary)	0.508	--	--	--	--	--
No Metastases	0.722	--	--	--	--	--
Other than chemotherapy	0.017	0.149	1.160	1.025	1.296	0.031
ECOG PS	0.955	--	--	--	--	--
Needs met	0.008	0.146	1.157	1.055	1.259	0.005
**Log-phi (SE)**		**-2.472 (0.063)**				

Variable	ROLE Function: Univariate analysis P-value	Multivariate analysis β	OR	95%CI		P-value
Intercept		-0.299	0.741	0.586	0.897	0.000
HQIS vs Usual Care		0.101	1.106	1.010	1.202	0.041
HQIS Implementation Epoch 1st vs 3rd		-0.023	0.977	0.852	1.102	0.717
HQIS Implementation Epoch 2nd vs 3rd		0.038	1.039	0.928	1.150	0.502
Age (yr)	0.454	--	--	--	--	--
Sex (male)	0.750	--	--	--	--	--
Married	0.263	--	--	--	--	--
Instruction (more primary)	0.845	--	--	--	--	--
No Metastases	0.182	-0.076	0.927	0.826	1.028	0.140
Other than chemotherapy	0.184	0.089	1.093	0.963	1.223	0.180
ECOG PS	0.032	0.117	1.124	1.013	1.235	0.040
Needs met	<0.001	0.193	1.213	1.115	1.311	0.000
**Log-phi (SE)**		**-2.573 (0.063)**				

Variable	COGNITIVE Function: Univariate analysis P-value	Multivariate analysis β	OR	95%CI		P-value
Intercept		-0.265	0.767	0.628	0.907	0.000
HQIS vs Usual Care		0.057	1.059	0.972	1.145	0.195
HQIS Implementation Epoch 1st vs 3rd		-0.023	0.977	0.865	1.090	0.690
HQIS Implementation Epoch 2nd vs 3rd		0.085	1.089	0.989	1.188	0.096
Age (yr)	0.992	--	--	--	--	--
Sex (male)	0.359	--	--	--	--	--
Married	0.478	--	--	--	--	--
Instruction (more primary)	0.477	--	--	--	--	--
No Metastases	0.199	-0.074	0.929	0.839	1.020	0.112
Other than chemotherapy	<0.001	0.210	1.234	1.118	1.350	0.000
ECOG PS	0.037	0.112	1.118	1.019	1.218	0.028
Needs met	0.003	0.151	1.163	1.075	1.251	0.001
**Log-phi (SE)**		**-2.573 (0.063)**				

Variable	Symptom FATIGUE: Univariate analysis P-value	Multivariate analysis β	OR	95%CI		P-value
Intercept		0.227	1.255	1.110	1.400	0.002
HQIS vs Usual Care		-0.097	0.908	0.818	0.998	0.035
HQIS Implementation Epoch 1st vs 3rd		0.015	1.015	0.899	1.132	0.799
HQIS Implementation Epoch 2nd vs 3rd		-0.077	0.926	0.822	1.029	0.145
Age (yr)	0.336	--	--	--	--	--
Sex (male)	0.179	--	--	--	--	--
Married	0.224	--	--	--	--	--
Instruction (more primary)	0.599	--	--	--	--	--
No Metastases	0.114	0.088	1.092	0.998	1.186	0.068
Other than chemotherapy	0.021	-0.150	0.861	0.740	0.981	0.015
ECOG PS	0.026	-0.117	0.890	0.786	0.994	0.028
Needs met	0.004	-0.159	0.853	0.762	0.944	0.001
**Log-phi (SE)**		**-2.376 (0.064)**				

Variable	Symptom NAUSEA: Univariate analysis P-value	Multivariate analysis β	OR	95%CI		P-value
Intercept		0.166	1.180	0.929	1.432	0.197
HQIS vs Usual Care		-0.063	0.939	0.854	1.023	0.143
HQIS Implementation Epoch 1st vs 3rd		-0.079	0.924	0.814	1.035	0.163
HQIS Implementation Epoch 2nd vs 3rd		-0.050	0.951	0.853	1.049	0.313
Age (yr)	0.082	0.000	1.000	0.996	1.003	0.789
Sex (male)	0.003	-0.090	0.914	0.829	0.999	0.039
Married	0.782	--	--	--	--	--
Instruction (more primary)	0.102	0.065	1.067	0.985	1.149	0.123
No Metastases	0.030	0.075	1.078	0.988	1.168	0.102
Other than chemotherapy	0.002	-0.171	0.843	0.729	0.957	0.003
ECOG PS	0.027	-0.080	0.923	0.823	1.022	0.113
Needs met	0.004	-0.138	0.871	0.785	0.957	0.002
**Log-phi (SE)**		**-2.910 (0.069)**				

Variable	Symptom PAIN: Univariate analysis P-value	Multivariate analysis β	OR	95%CI		P-value
Intercept		0.132	1.142	0.880	1.403	0.321
HQIS vs Usual Care		-3.69E-04	1.000	0.905	1.094	0.994
HQIS Implementation Epoch 1st vs 3rd		0.019	1.019	0.897	1.142	0.762
HQIS Implementation Epoch 2nd vs 3rd		0.005	1.005	0.897	1.114	0.924
Age (yr)	0.186	-0.002	0.998	0.995	1.002	0.315
Sex (male)	0.604	--	--	--	--	--
Married	0.387	--	--	--	--	--
Instruction (more primary)	0.514	--	--	--	--	--
No Metastases	0.076	0.084	1.087	0.989	1.186	0.095
Other than chemotherapy	0.754	--	--	--	--	--
ECOG PS	0.231	--	--	--	--	--
Needs met	0.118	-0.078	0.925	0.828	1.022	0.114
**Log-phi (SE)**		**-2.593 (0.063)**				

Variable	Symptom DYSPNOEA: Univariate analysis P-value	Multivariate analysis β	OR	95%CI		P-value
Intercept		-0.084	0.919	0.763	1.076	0.293
HQIS vs Usual Care		-0.137	0.872	0.757	0.986	0.019
HQIS Implementation Epoch 1st vs 3rd		-0.085	0.919	0.772	1.065	0.257
HQIS Implementation Epoch 2nd vs 3rd		-0.072	0.930	0.798	1.062	0.283
Age (yr)	0.812	--	--	--	--	--
Sex (male)	0.755	--	--	--	--	--
Married	0.527	--	--	--	--	--
Instruction (more primary)	0.903	--	--	--	--	--
No Metastases	0.246	--	--	--	--	--
Other than chemotherapy	0.283	--	--	--	--	--
ECOG PS	0.258	--	--	--	--	--
Needs met	0.022	-0.153	0.858	0.741	0.975	0.011
**Log-phi (SE)**		**-2.147 (0.062)**				

Variable	Symptom INSOMNIA: Univariate analysis P-value	Multivariate analysis β	OR	95%CI		P-value
Intercept		-0.002	0.998	0.689	1.308	0.992
HQIS vs Usual Care		-0.021	0.979	0.866	1.091	0.711
HQIS Implementation Epoch 1st vs 3rd		-0.105	0.901	0.753	1.048	0.164
HQIS Implementation Epoch 2nd vs 3rd		-0.221	0.802	0.672	0.931	0.001
Age (yr)	0.200	0.004	1.004	1.000	1.008	0.052
Sex (male)	0.108	-0.080	0.923	0.809	1.037	0.170
Married	0.862	--	--	--	--	--
Instruction (more primary)	0.492	--	--	--	--	--
No Metastases	0.097	0.106	1.112	0.994	1.230	0.077
Other than chemotherapy	0.369	--	--	--	--	--
ECOG PS	0.321	--	--	--	--	--
Needs met	0.004	-0.174	0.840	0.725	0.955	0.003
**Log-phi (SE)**		**-2.180 (0.061)**				

Variable	Symptom APPETITE LOSS: Univariate analysis P-value	Multivariate analysis β	OR	95%CI		P-value
Intercept		0.269	1.309	1.136	1.482	0.002
HQIS vs Usual Care		-0.074	0.928	0.815	1.042	0.199
HQIS Implementation Epoch 1st vs 3rd		-0.042	0.959	0.812	1.106	0.574
HQIS Implementation Epoch 2nd vs 3rd		-0.117	0.890	0.760	1.019	0.076
Age (yr)	0.575	--	--	--	--	--
Sex (male)	0.213	--	--	--	--	--
Married	0.990	--	--	--	--	--
Instruction (more primary)	0.673	--	--	--	--	--
No Metastases	0.101	0.126	1.135	1.019	1.251	0.033
Other than chemotherapy	0.076	-0.144	0.866	0.714	1.018	0.064
ECOG PS	0.213	--	--	--	--	--
Needs met	<0.001	-0.264	0.768	0.653	0.884	<0.001
**Log-phi (SE)**		**-2.189 (0.063)**				

Variable	Symptom CONSTIPATION: Univariate analysis P-value	Multivariate analysis β	OR	95%CI		P-value
Intercept		0.197	1.217	1.100	1.334	0.001
HQIS vs Usual Care		0.016	1.016	0.913	1.119	0.763
HQIS Implementation Epoch 1st vs 3rd		-0.094	0.910	0.777	1.044	0.169
HQIS Implementation Epoch 2nd vs 3rd		-0.174	0.840	0.721	0.960	0.004
Age (yr)	0.223	--	--	--	--	--
Sex (male)	0.202	--	--	--	--	--
Married	0.628	--	--	--	--	--
Instruction (more primary)	0.727	--	--	--	--	--
No Metastases	0.259	--	--	--	--	--
Other than chemotherapy	0.021	-0.175	0.839	0.700	0.979	0.014
ECOG PS	0.944	--	--	--	--	--
Needs met	0.475	--	--	--	--	--
**Log-phi (SE)**		**-2.375 (0.062)**				

Variable	Symptom INSOMNIA: Univariate analysis P-value	Multivariate analysis β	OR	95%CI		P-value
Intercept		-0.002	0.998	0.689	1.308	0.992
HQIS vs Usual Care		-0.021	0.979	0.866	1.091	0.711
HQIS Implementation Epoch 1st vs 3rd		-0.105	0.901	0.753	1.048	0.164
HQIS Implementation Epoch 2nd vs 3rd		-0.221	0.802	0.672	0.931	0.001
Age (yr)	0.200	0.004	1.004	1.000	1.008	0.052
Sex (male)	0.108	-0.080	0.923	0.809	1.037	0.170
Married	0.862	--	--	--	--	--
Instruction (more primary)	0.492	--	--	--	--	--
No Metastases	0.097	0.106	1.112	0.994	1.230	0.077
Other than chemotherapy	0.369	--	--	--	--	--
ECOG PS	0.321	--	--	--	--	--
Needs met	0.004	-0.174	0.840	0.725	0.955	0.003
**Log-phi (SE)**		**-2.180 (0.061)**				

Variable	Symptom APPETITE LOSS: Univariate analysis P-value	Multivariate analysis β	OR	95%CI		P-value
Intercept		0.269	1.309	1.136	1.482	0.002
HQIS vs Usual Care		-0.074	0.928	0.815	1.042	0.199
HQIS Implementation Epoch 1st vs 3rd		-0.042	0.959	0.812	1.106	0.574
HQIS Implementation Epoch 2nd vs 3rd		-0.117	0.890	0.760	1.019	0.076
Age (yr)	0.575	--	--	--	--	--
Sex (male)	0.213	--	--	--	--	--
Married	0.990	--	--	--	--	--
Instruction (more primary)	0.673	--	--	--	--	--
No Metastases	0.101	0.126	1.135	1.019	1.251	0.033
Other than chemotherapy	0.076	-0.144	0.866	0.714	1.018	0.064
ECOG PS	0.213	--	--	--	--	--
Needs met	<0.001	-0.264	0.768	0.653	0.884	<0.001
**Log-phi (SE)**		**-2.189 (0.063)**				

Variable	Symptom CONSTIPATION: Univariate analysis P-value	Multivariate analysis β	OR	95%CI		P-value
Intercept		0.197	1.217	1.100	1.334	0.001
HQIS vs Usual Care		0.016	1.016	0.913	1.119	0.763
HQIS Implementation Epoch 1st vs 3rd		-0.094	0.910	0.777	1.044	0.169
HQIS Implementation Epoch 2nd vs 3rd		-0.174	0.840	0.721	0.960	0.004
Age (yr)	0.223	--	--	--	--	--
Sex (male)	0.202	--	--	--	--	--
Married	0.628	--	--	--	--	--
Instruction (more primary)	0.727	--	--	--	--	--
No Metastases	0.259	--	--	--	--	--
Other than chemotherapy	0.021	-0.175	0.839	0.700	0.979	0.014
ECOG PS	0.944	--	--	--	--	--
Needs met	0.475	--	--	--	--	--
**Log-phi (SE)**		**-2.375 (0.062)**				

Variable	Symptom DIARRHEA: Univariate analysis P-value	Multivariate analysis β	OR	95%CI		P-value
Intercept		0.097	1.101	0.966	1.237	0.164
HQIS vs Usual Care		-0.021	0.979	0.897	1.060	0.607
HQIS Implementation Epoch 1st vs 3rd		0.012	1.013	0.906	1.119	0.819
HQIS Implementation Epoch 2nd vs 3rd		-0.034	0.967	0.872	1.062	0.489
Age (yr)	0.965	--	--	--	--	--
Sex (male)	0.165	-0.038	0.963	0.882	1.045	0.368
Married	0.545	--	--	--	--	--
Instruction (more primary)	0.834	--	--	--	--	--
No Metastases	0.059	0.058	1.059	0.972	1.146	0.193
Other than chemotherapy	0.525	--	--	--	--	--
ECOG PS	0.006	-0.119	0.888	0.792	0.983	0.015
Needs met	0.164	-0.068	0.935	0.851	1.018	0.114
**Log-phi (SE)**		**-2.971 (0.070)**				

Variable	Symptom FINANCIAL PROBLEMS: Univariate analysis P-value	Multivariate analysis β	OR	95%CI		P-value
Intercept		0.182	1.200	1.049	1.351	0.018
HQIS vs Usual Care		0.012	1.012	0.922	1.103	0.787
HQIS Implementation Epoch 1st vs 3rd		-0.086	0.918	0.801	1.035	0.151
HQIS Implementation Epoch 2nd vs 3rd		-0.093	0.911	0.807	1.015	0.079
Age (yr)	0.214	--	--	--	--	--
Sex (male)	0.015	-0.096	0.909	0.821	0.997	0.033
Married	0.126	0.064	1.066	0.966	1.166	0.212
Instruction (more primary)	0.583	--	--	--	--	--
No Metastases	0.345	--	--	--	--	--
Other than chemotherapy	0.084	-0.102	0.903	0.782	1.023	0.096
ECOG PS	0.686	--	--	--	--	--
Needs met	0.002	-0.127	0.881	0.789	0.973	0.007
**Log-phi (SE)**		**-2.749 (0.067)**				

© 2021 Caminiti C et al. *JAMA Network Open.*

eTable 4. Effect of HQIS on mood disorder measured with the HADS-D. Regression coefficient (β), Odds Ratio (OR) and 95% confidence intervals (CI) of the variables included in the analysis, and P-value of the General Linear Model.

	**GLM Model - MOOD**						
	**Univariate analysis**		**Multivariate analysis**				
**Variable**	**P-value**		**β**	**SE**	**95%CI**		**P-value**
Intercept			*8.354*	*1.042*	*6.311*	*10.396*	*<0.001*
HQIS vs Usual Care			***0.084***	***0.353***	***-0.607***	*0.776*	***0.811***
HQIS Implementation Epoch 1st vs 3rd			*-1.708*	*0.455*	*-2.599*	*-0.816*	*<0.001*
HQIS Implementation Epoch 2nd vs 3rd			*0.074*	*0.404*	*-0.718*	*0.865*	*0.855*
Age (yr)	0.096		*0.004*	*0.013*	-0.021	0.029	*0.764*
Sex (male)	0.962						
Married	0.266						
Instruction (more primary)	<0.001		-0.967	0.340	-1.632	-0.301	0.005
No Metastases	0.069		-0.772	0.370	-1.497	-0.047	0.037
Other than chemotherapy	0.001		-1.151	0.470	-2.073	-0.230	0.015
ECOG PS	0.003		0.983	0.412	0.175	1.791	0.017
Needs met	<0.001		-0.960	0.383	-0.313	1.079	0.007
**AIC**			**3638.4**				

© 2021 Caminiti C et al. *JAMA Network Open.*

eTable 5. Results of the subgroup analysis aimed to identify any effect modifiers on the emotional function of HRQoL. Regression coefficient (β), Odds Ratio (OR) and 95% confidence intervals (CI) of the interaction term, and P-value of the Binomial Beta Regression Model.

COEFF. OF INTERACTION TERM	β	OR	95% CI	P-value
Age (yr)	0.000	1.000	*(0.993-1.006)*	0.899
Age < 52 (*1st* quartile)	-0.010	0.991	*(0.798-1.183)*	0.923
Age ≥ 71 (*3rd* quartile)	0.046	1.047	*(0.854-1.239)*	0.643
Sex (male)	-0.014	0.986	*(0.81-1.162)*	0.875
Not Married	0.098	1.103	*(0.900-1.305)*	0.345
Primary education or less	0.087	1.090	*(0.923-1.258)*	0.310
High school	-0.110	0.896	*(0.727-1.065)*	0.203
Graduate School	0.058	1.060	*(0.795-1.324)*	0.667
Instruction (more primary)	*-0.087*	*0.917*	*(0.75-1.084)*	*0.310*
Metastases	0.073	1.075	(0.892-1.259)	0.437
Chemotherapy	-0.042	0.959	*(0.711-1.207)*	0.740
ECOG PS	0.057	1.059	*(0.973-1.145)*	0.191
ECOG PS (=0)	0.075	1.078	*(0.875-1.28)*	0.470
HADS A ≥ 9	0.058	1.059	*(0.882-1.237)*	0.524
HADS D ≥ 8	-0.002	0.998	*(0.823-1.174)*	0.985
Needs<br>(sum > 6 median value)	-0.089	0.915	*(0.745-1.084)*	0.301
Sum Needs	-0.001	0.999	*(0.985-1.014)*	0.921

© 2021 Caminiti C et al. *JAMA Network Open.*
