# Supplementary material for: Effectiveness of a Psychosocial Care Quality Improvement Strategy to Address Quality of Life in Patients With Cancer: The HuCare2 Stepped-Wedge Cluster Randomized Trial
Source: JAMA Netw Open. 2021 Oct 14;4(10):e2128667. doi: 10.1001/jamanetworkopen.2021.28667 (PMC8517739; doi:10.1001/jamanetworkopen.2021.28667)
Supplement: Supplement 3. — Data Sharing Statement [file jamanetwopen-e2128667-s003.pdf]

# Data Sharing Statement

Caminiti. Effectiveness of a Psychosocial Care Quality Improvement Strategy to Address Quality of Life in Patients With Cancer. *JAMA Netw Open*. Published October 14, 2021. doi:10.1001/jamanetworkopen.2021.28667

**Data**
**Data available:** No

**Additional Information**
**Explanation for why data not available:** Data available on request only for aggregate use in meta-analyses
